# ppGpp influences protein protection, growth and photosynthesis in *Phaeodactylum tricornutum*

**DOI:** 10.1111/nph.17286

**Published:** 2021-03-19

**Authors:** Luisana Avilan, Regine Lebrun, Carine Puppo, Sylvie Citerne, Stephane Cuiné, Yonghua Li‐Beisson, Benoît Menand, Ben Field, Brigitte Gontero

**Affiliations:** ^1^ CNRS BIP UMR 7281 IMM FR 3479 Aix Marseille Univ 31 Chemin Joseph Aiguier Marseille 13009 France; ^2^ Centre for Enzyme Innovation School of Biological Sciences Institute of Biological and Biomedical Sciences University of Portsmouth Portsmouth PO1 2DY UK; ^3^ Plate‐forme Protéomique Marseille Protéomique (MaP) IMM FR 3479, 31 Chemin Joseph Aiguier Marseille 13009 France; ^4^ Institut Jean‐Pierre Bourgin INRAE AgroParisTech Université Paris‐Saclay Versailles 78000 France; ^5^ CEA, CNRS, UMR7265 BIAM CEA Cadarache Aix‐Marseille Univ Saint‐Paul‐lez Durance 13108 France; ^6^ CEA, CNRS, UMR7265 BIAM Aix‐Marseille Univ Marseille 13009 France

**Keywords:** chloroplast, diatoms, lipid droplets, *Phaeodactylum tricornutum*, photosynthesis, ppGpp, proteome

## Abstract

- Chloroplasts retain elements of a bacterial stress response pathway that is mediated by the signalling nucleotides guanosine penta‐ and tetraphosphate ((p)ppGpp). In the model flowering plant Arabidopsis, ppGpp acts as a potent regulator of plastid gene expression and influences photosynthesis, plant growth and development. However, little is known about ppGpp metabolism or its evolution in other photosynthetic eukaryotes.
- Here, we studied the function of ppGpp in the diatom *Phaeodactylum tricornutum* using transgenic lines containing an inducible system for ppGpp accumulation. We used these lines to investigate the effects of ppGpp on growth, photosynthesis, lipid metabolism and protein expression.
- We demonstrate that ppGpp accumulation reduces photosynthetic capacity and promotes a quiescent‐like state with reduced proliferation and ageing. Strikingly, using nontargeted proteomics, we discovered that ppGpp accumulation also leads to the coordinated upregulation of a protein protection response in multiple cellular compartments.
- Our findings highlight the importance of ppGpp as a fundamental regulator of chloroplast function across different domains of life, and lead to new questions about the molecular mechanisms and roles of (p)ppGpp signalling in photosynthetic eukaryotes.

## Introduction

Diatoms are a group of unicellular eukaryotic photosynthetic organisms that form a major part of phytoplankton, and are responsible for up to one‐fifth of net global carbon fixation (Falkowski *et al*., 2000). Beside their ecological importance, diatoms are also studied because they have a wide range of potential applications that include drug delivery in chemotherapy, biofuels and as environmental indicators for monitoring water quality (Levitan *et al*., 2014; Lavoie *et al*., 2017; Uthappa *et al*., 2018).

Diatoms appeared relatively recently in evolutionary history, around 200 million years ago (Medlin, 2016; Benoiston *et al*., 2017). The diatom chloroplast was acquired through complex endosymbiotic events, where it is thought that a red algal ancestor was engulfed by a eukaryote that already possessed green algal genes from a previous endosymbiosis (Dorrell *et al*., 2017). The acquisition of additional genes by lateral gene transfer from bacteria is also likely to have been an important driving force in the evolution of diatoms (Bowler *et al*., 2008). These complex origins confer a unique cellular physiology to diatoms that allows them to adapt to multiple environments (Vardi *et al*., 2008; Gruber & Kroth, 2017).

The diatom chloroplast differs in a number of aspects from the chloroplast of plants and green algae. At the level of membrane architecture, the diatom chloroplast is surrounded by four membranes, and the thylakoids are loosely stacked with three interconnected membranes (Flori *et al*., 2017). Moreover, the light‐harvesting complex (LHC) of diatoms is known as the fucoxanthin–chlorophyll protein complex (FCP), and is composed of tetramers containing Chl*c* and the carotenoid fucoxanthin (Lepetit *et al*., 2012; Roding *et al*., 2018; Nagao *et al*., 2019; Pi *et al*., 2019). Diatoms have other specific features such as a functional urea cycle (Allen *et al*., 2011) and a eukaryotic Entner–Doudoroff glycolytic pathway (Fabris *et al*., 2012), and they lack the plastid oxidative pentose phosphate pathway found in plants (Gruber & Kroth, 2017). The regulation of diatom metabolism is also distinct from that in plants (Jensen *et al*., 2017; Launay *et al*., 2020).

Like plants, diatoms possess signalling pathways with prokaryotic origins such as the bacterial histidine‐kinase‐based two‐component systems (Bowler *et al*., 2008), as well as pathways of eukaryotic origins such as the target of rapamycin (TOR) kinase (Prioretti *et al*., 2017) and the G protein‐coupled receptor signalling pathway (Port *et al*., 2013).

Another signalling pathway that could play an important role in diatom stress acclimation is the pathway mediated by the nucleotides guanosine tetraphosphate and guanosine pentaphosphate ((p)ppGpp) (Field, 2018; Avilan *et al*., 2019; Prioretti *et al*., 2020). In bacteria, ppGpp and, to a lesser extent, pppGpp accumulate in response to a range of different stresses, and specifically target transcription and translation to slow growth and promote stress acclimation (Hauryliuk *et al*., 2015; Steinchen & Bange, 2016). (p)ppGpp is also found in plants and green algae (Takahashi *et al*., 2004), and chloroplast‐targeted enzymes related to the *E. coli* RelA / SpoT (p)ppGpp synthetases are widespread among the photosynthetic eukaryotes (Atkinson *et al*., 2011; Ito *et al*., 2017; Avilan *et al*., 2019). Currently, the function of ppGpp is best characterized in the flowering plant Arabidopsis, where it inhibits chloroplast gene expression, reduces chloroplast size and reduces photosynthetic capacity (Maekawa *et al*., 2015; Sugliani *et al*., 2016; Honoki *et al*., 2018). While the mechanisms are still uncertain, ppGpp may act by downregulating the transcription of chloroplast‐encoded genes via the inhibition of chloroplastic RNA polymerases or GTP biosynthesis (Nomura *et al*., 2014; Yamburenko *et al*., 2015; Sugliani *et al*., 2016). Concentrations of ppGpp increase in response to different abiotic stresses, as well as in response to treatment with stress‐associated plant hormones (Takahashi *et al*., 2004). Increased ppGpp concentrations can affect growth under nitrogen‐limiting conditions (Maekawa *et al*., 2015; Honoki *et al*., 2018) and plant immune signalling (Abdelkefi *et al*., 2018).

Genes coding for RelA SpoT Homolog (RSH) enzymes are present in all fully sequenced photosynthetic eukaryotes (Field, 2018; Avilan *et al*., 2019). However, very little is known about the role of (p)ppGpp in algae, and in particular in algae of the red lineage. A recent study in the extremophile red alga *Cyanidioschyzon merolae* showed that overexpression of CmRSH4b, a functional (p)ppGpp synthetase, results in a reduction in chloroplast size and decreased chloroplast rRNA transcription, although (p)ppGpp concentrations were not measured (Imamura *et al*., 2018). The situation in diatoms, with their mixed red‐green heritage and specific lifestyles, is even less clear. A recent analysis showed that the nuclear genome of the marine diatom *Phaeodactylum tricornutum* encodes three functional RSH enzymes from red lineage‐specific clades: PtRSH1, a bifunctional (p)ppGpp synthetase/hydrolase; and PtRSH4a and PtRSH4b, which act exclusively as (p)ppGpp synthetases (Avilan *et al*., 2019). Here, we studied (p)ppGpp signalling in *P. tricornutum* by manipulating endogenous ppGpp concentrations using transgenic lines that express a bacterial (p)ppGpp synthetase under the control of an inducible promoter. The use of a distantly related bacterial enzyme reduces the possibility of regulatory feedback that could occur using endogenous RSH enzymes. We found that ppGpp accumulation led to a reduction in photosynthetic capacity and an inhibition of ageing and growth. Strikingly, a proteomic analysis revealed that ppGpp accumulation also leads to the robust activation of a protein protection response involving chaperones and proteases. Our findings demonstrate that core ppGpp signalling is highly conserved across the photosynthetic eukaryotes, and that ppGpp has species‐specific roles that may be linked to adaptation to particular environments and lifestyles.

## Materials and Methods

### Cell culture

*Phaeodactylum tricornutum* Bohlin (strain name Pt1_8.6; RCC 2967) was obtained from Roscoff Culture Collection. Cells were grown in f/2 medium (Guillard & Ryther, 1962) without silica and adjusted to pH 8. The medium contained either 0.88 mM NaNO_3_ (f/2‐NO_3_) or NH_4_Cl (f/2‐NH_4_) as a nitrogen source. Cultures in liquid medium in Erlenmeyer flask or on agar plates (2% bacto‐agar) were maintained at 18°C under continuous illumination (30 µmol photon m^−2^ s^−1^) with LED lamps. Samples for cell counting (Malassez counting chamber) and protein extraction were withdrawn from liquid cultures.

### Plasmid construction and transformation of *P. tricornutum*

The vector pPha‐NR carrying the nitrate reductase promoter and the *ble* gene for zeocin resistance (Chu *et al*., 2016) (GenBank accession number JN180663, kindly provided by Prof. P. Kroth), was used as backbone for all plasmid constructions. The DNA fragments used for gene fusion and cloning in the final constructs were amplified by PCR using Q5 DNA polymerase (New England Biolabs, Evry, France) under standard conditions. The primers are specified in Supporting Information Table S1. The amplified fragments were assembled in the vector, previously digested with *Eco*RI and *Hind*III, by the sequence and ligation independent cloning (SLIC) method (Jeong *et al*., 2012).

The genes coding for a (p)ppGpp synthetase (SYN) and an inactive mutant form (D275G) of this synthetase (SYN^D>G^) were obtained by PCR, using plasmids described in Sugliani *et al*. (2016) as templates. SYN corresponds to the (p)ppGpp synthetase RelA from *E. coli* (residue 1 to 386). To target the enzymes to the chloroplast, the genes were fused by PCR to the sequence coding for the bipartite targeting sequence of the chloroplastic gamma ATP synthetase of *P. tricornutum* (GeneBank accession number U29898) whose ability to target proteins to the chloroplast is well characterized (Apt *et al*., 2002; Liu *et al*., 2016). A Kozak sequence (AAG) was included in the primer before the ATG translation start site to facilitate translation. The final constructions were used to transform cells by particle bombardment as previously described (Falciatore *et al*., 1999; Kroth, 2007). Full details of the transformation procedure are provided in Methods S1. Multiple independent transgenics lines were used for each construction in subsequent experiments.

### Nucleotide determination

**Fig. 1:**
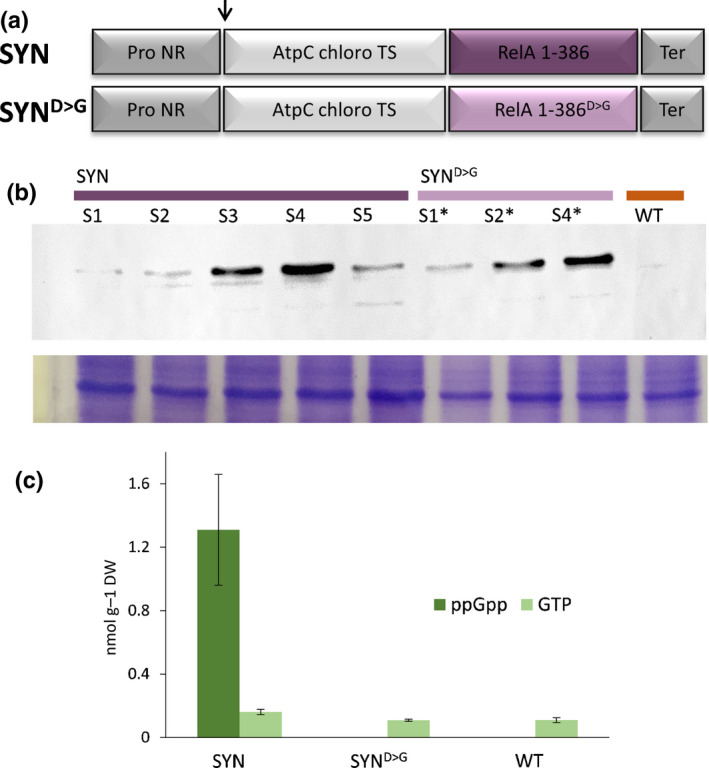
The creation of nitrate‐inducible lines for the accumulation of guanosine penta‐ and tetraphosphate (ppGpp) in *Phaeodactylum tricornutum*. (a) Schematic representation of the construction used for the transformation of *P. tricornutum* cells. The nitrate reductase promoter (Pro NR) and terminator (Term) were from the pPha‐NR vector. The bipartite targeting sequence was from the chloroplastic gamma ATP synthase (AtpC chloro TS). The genes encode a (p)ppGpp synthetase (SYN) and an inactive synthetase (SYN^D>G^). (b) Western blot analysis of extracts from independent SYN (S1–S5) and SYN^D>G^ (S1*–S4*) transgenic lines and the wild‐type (WT) grown in inducing f/2‐NO_3_ media using anti‐RelA antibodies specific for the RelA domain in SYN and SYN^D>G^. Each well was loaded with 7 × 10^4^ cells. Coomassie blue staining is shown as a loading control. (c) At 2 d after transfer to inducing f/2‐NO_3_ media, ppGpp and guanosine triphosphate (GTP) concentrations were determined in SYN (±SE, *n* = 5 independent lines), SYN^D>G^ (±SE, *n* = 4 independent lines) and WT lines (±SE, *n* = 3 independent replicates).

Guanosine triphosphate (GTP) and ppGpp concentrations were determined using stable isotope labelled internal standards as described (Bartoli *et al*., 2020). Briefly, 8 mg (DW equivalent) of *P. tricornutum* cells were harvested from cultures at 2 d after induction and nucleotides were directly extracted with 3 ml 2 M formic acid containing 12.5 pmol ^13^C‐labelled ppGpp and 125 pmol ^13^C‐labelled GTP. Extracts were incubated on ice for 30 min, then 3 ml 50 mM ammonium acetate at pH 4.5 was added; samples were then loaded onto pre‐equilibrated Oasis WAX SPE cartridges and nucleotides were eluted with a mixture of methanol/water/NH_4_OH (20 : 70 : 10). The eluates were lyophilized and resuspended in water before analysis by high‐performance liquid chromatography (HPLC)‐MS/MS with multiple reaction monitoring. The quantification of (p)ppGpp in Fig. 1(c) was hindered by poor detection of the ^13^C‐ppGpp internal standard during HPLC‐MS/MS analysis. ppGpp concentrations were therefore calculated using the ^13^C‐GTP internal standard adjusted for the response factor to estimate recovery. Fig. S1 shows absolute quantification of ppGpp based on the ^13^C‐ppGpp internal standard.

### Growth parameters and pigment quantification

For the analysis of growth, phenotype cells were grown in 25 ml liquid f/2‐NH_4_ in 250 ml Erlenmeyer flasks, without agitation, in triplicate for five different transformants. Optical density (OD) at 750 nm and number of cells were monitored every 24 h. Cell number showed a linear relationship with OD 750 nm up to an OD of 1. Cultures were inoculated from a preculture to an initial OD 750 nm of 0.05. After 48 h, cells were centrifuged at 5000 ***g*** (Allegra^®^ X‐15R Centrifuge; Beckman Coulter) for 10 min at 16°C and the medium was substituted with f/2‐NO_3_ to induce the expression of SYN or SYN^D>G^. For control cultures, the medium was substituted with fresh f/2‐NH_4_. Pigments were extracted on ice from cell pellets (1.6 × 10^7^ cells) with 1 ml 96% (v/v) ethanol, maintained in the dark for 30 min and centrifuged at 13 000 ***g*** for 10 min at 4°C. The spectra of the supernatant from 350 to 750 nm were recorded using a PTP‐6 Peltier System spectrophotometer (Perkin Elmer, Waltham, MA, USA). Chlorophyll *a* and *c* concentrations were calculated according to Ritchie (2006) and fucoxanthin concentration according to Wang *et al*. (2018). For extended darkness, at 24 h after induction cells were transferred to darkness for 21 d.

### Light microscopy and cell measurement

Light microscopy images of live cells were taken using a Motic BA410 microscope equipped with a Moticam 1080 camera (Motic, Barcelona, Spain). Images were used to determine the length and width of individual cells using the motic image 3 plus software after calibration of the microscope. For fluorescence microscopy, cells were incubated with the fluorescent dye AC202 as described in Harchouni *et al*. (2018). Fluorescence was visualized in an epifluorescence microscope Eclipse 80i (Nikon) using the DAPI filter cube (excitation filter, 360BP40; emission filter, 460BP50). Images were acquired using ms elements imaging software (Nikon, Melville, NY, USA). Images were merged using imagej software.

### Electron microscopy and immunogold labelling

Procedures for electron microscopy and immunogold labelling are described in Methods S1.

### Measurement of photosynthetic activity

Chlorophyll fluorescence parameters were measured using a Fluorcam FC 800‐O imaging fluorometer (Photon System Instruments, Drasov, Czech Republic). Cells plated on solid medium were recovered in f2/NH_4_ medium and 10 µl of this cell suspension (between 5 and 9 × 10^7^ cells ml^−1^) was dotted onto f2/NO_3_ or f2/NH_4_ medium and grown under standard growth conditions. Before measurement, the plates were kept in the dark for 20 min and the Chl fluorescence was imaged to obtain minimum (*F*_o_) and maximum fluorescence yield (*F*_m_). photosystem II (PSII) maximum efficiency was calculated as *F*_v_/*F*_m_ = (*F*_m_ − *F*_o_)/*F*_m_. To calculate relative electron transfer rate (rETR), samples were exposed to different photon flux densities (PPFD) in a stepwise fashion. Relative ETR was then calculated as the product of the photochemical yield of PSII (ΦP = Δ*F*/*F*_m_′ = (*F*_m_′ − *F*_o_)/*F*_m_′) and PPFD.

### Cell extract, SDS‐PAGE and immunoblotting

*Phaeodactylum tricornutum* cells (2 × 10^7^) were harvested from liquid culture by centrifugation (5000 ***g*** for 15 min at 18°C) and resuspended in 100 µl of rupture buffer (10 mM Tris, pH 8 containing 2% n‐dodecyl β‐d‐maltoside) and then broken by sonication using six pulses from an ultrasonicator (Sonics & Materials Inc., Vibracell, Bioblock, Danbury, CT, USA). After incubation at 4°C for 30 min, the cell extract was centrifuged at 12 000 ***g*** for 30 min at 4°C. The supernatant was submitted to sodium dodecyl sulphate–polyacrylamide gel electrophoresis (SDS‐PAGE) and gels were stained with Coomassie blue R‐250. For Western blot analysis, proteins were transferred onto nitrocellulose membrane as previously described (Sambrook *et al*., 1989). Loading control gels were run in parallel. The membrane was blocked with TBS (Tris 10 mM, pH 8, 150 mM NaCl) containing 5% nonfat milk and incubated with the specified primary antibodies in the same buffer containing 1% nonfat milk. The antibodies used were as follows: anti‐*E.coli* RelA (dilution 1 : 2000, kindly provided by M. Cashel), anti‐haemagglutinin (HA) (monoclonal, dilution 1 : 10 000; H9658, clone HA‐7; Sigma‐Aldrich), anti‐PsbA (polyclonal, dilution 1 : 10 000; AS05 084; Agrisera) and anti‐*P. tricornutum* LHCf1‐LHCf11 (Juhas and Buchel, 2012) (polyclonal, dilution 1 : 5000, kindly provided by C. Büchel). After washing with TBS, the membranes were incubated with either horseradish peroxidase;coupled anti‐rabbit IgG (NA934V; GE Healthcare, Pittsburg, PA, USA) for the polyclonal antibodies or anti‐mouse IgG (Sigma‐Aldrich) for the monoclonal antibodies. Immunodetection was performed using the enhanced chemiluminescence method (ECL substrates, GE Healthcare).

### Lipid and chrysolaminarin determination

The fluorescent probe Nile red was used to detect neutral lipids by flow cytometry as specified in Prioretti *et al*. (2017). The cells at different growth phases were fixed with 2% (v/v) glutaraldehyde (Prioretti *et al*., 2017) and 1 ml of cell suspension was incubated with 2 µl of Nile red solution (0.25 mg ml^−1^) for 5 min before the analysis with a bench flow cytometer (BD Accuri C6; BD Biosciences, San Jose, CA, USA). To determine Nile red fluorescence, the trigger signal was set to FL2 fluorescence and combined with the side scatter signal. Ten thousand events were analysed in each case.

For lipid analysis, cells of *P. tricornutum* (50 × 10^6^ cells) were harvested by centrifugation (5000 ***g***, for 10 min at 4°C) and the pellet was resuspended with 1 ml of hot isopropanol (preheated at 85°C) and incubated at this temperature for 10 min to quench endogenous lipases. Detailed lipid extraction protocol can be found in Legeret *et al*. (2016). Extracted total lipids were then dried under a stream of N_2_ and then dissolved in a solvent mixture of chloroform: methanol (2 : 1, v/v) for quantification by thin layer chromatography (TLC). Two types of TLC were run: one for triacylglycerol (TAG) quantification, and a second for polar membrane lipid analysis. Detailed TLC procedures, standard used and quantifications have been described, together with total fatty acid (FA) content and composition analysis in Siaut *et al*. (2011).

Extraction and determination of the β‐1,3‐glucan chrysolaminarin were performed following the method of Granum & Myklestad (2002) from pellets containing 2 × 10^7^ cells. A calibration curve was obtained using glucose as a standard.

### Mass spectrometry analysis

Protein extracts (50 µg protein) from three SYN lines, two SYN^D>G^ lines and one WT, on equal numbers of cells, were loaded onto a gel (SDS‐PAGE) and the band from the stacking gel corresponding to the total proteins was excised and submitted to in‐gel trypsin digestion for proteomic analysis as previously described (Santin *et al*., 2018), with minor modifications. In parallel, total proteins were also separated to analyse the protein profile in the gel (Fig. S2). Tryptic peptide samples were quantified by a colorimetric peptide assay (Pierce, Thermo Fisher Scientific, Waltham, MA, USA) and aliquots of 200 ng were injected by LC‐MS/MS. Two LC‐MS/MS injections were performed per condition (technical replicates). Spectral data were processed for protein identification and quantification using the MaxQuant computational proteomics platform (v.1.6.5.0) integrating the search engine Andromeda and the maxlfq algorithm (Cox *et al*., 2014). The results are illustrated in a volcano plot (Fig. S3), and the list of identified proteins and differentially accumulating proteins in SYN vs the control lines is available in Table S2. Full details of LC‐MS/MS analysis conditions and quantification are provided in Methods S1.

## Results

### An inducible (p)ppGpp synthetase efficiently increases ppGpp concentrations in *P. tricornutum*

As a first step towards understanding the function of (p)ppGpp in diatoms, we developed a strategy similar to that reported in Arabidopsis (Sugliani *et al*., 2016), where (p)ppGpp accumulation was triggered using a constitutively active fragment of a bacterial (p)ppGpp synthetase. For this purpose, we transformed *P. tricornutum* with a chimeric gene (SYN) encoding the chloroplast targeting peptide of the chloroplastic gamma ATP synthetase (Apt *et al*., 2002; Liu *et al*., 2016) and a constitutively active fragment of the (p)ppGpp synthetase RelA (Schreiber *et al*., 1991) (Fig. 1a). SYN was placed under the control of an NO_3_‐inducible promoter. Control transgenic lines were also generated that express catalytically inactive forms of the same enzyme, SYN^D>G^. Multiple independent positive transformants were identified by PCR and analysed to confirm protein expression following induction by transfer from NH_4_‐containing medium to NO_3_‐containing medium (Fig. 1b). We confirmed that the SYN protein was targeted to the chloroplast by immunogold labelling (Fig. S4). Importantly, induction of SYN lines also caused an increase in ppGpp concentrations from below the limits of detection to 1.30 ± 0.34 nmol mg^−1^ dry cell weight (*n* = 5 independent biological replicates) (Fig. 1c). pppGpp was not detected, suggesting that SYN preferentially acts as a ppGpp synthetase, and/or that pppGpp can be converted to ppGpp by endogenous enzymes as in bacteria. No ppGpp was detected in SYN^D>G^ lines or the wild‐type (WT) under inducing conditions. The absence of basal concentrations of ppGpp is likely to be a consequence of the nutrient shift used for induction because we were able to detect low quantities of ppGpp in WT cells grown in standard media in the light (Fig. S1). Interestingly, we also observed that incubation of cells in the dark for prolonged periods led to the accumulation of ppGpp. We simultaneously quantified GTP concentrations and found that they were similar to the WT control regardless of ppGpp concentrations (Figs 1c, S1).

### ppGpp accumulation strongly inhibits cell division and has a major effect on photosynthesis

**Fig. 2:**
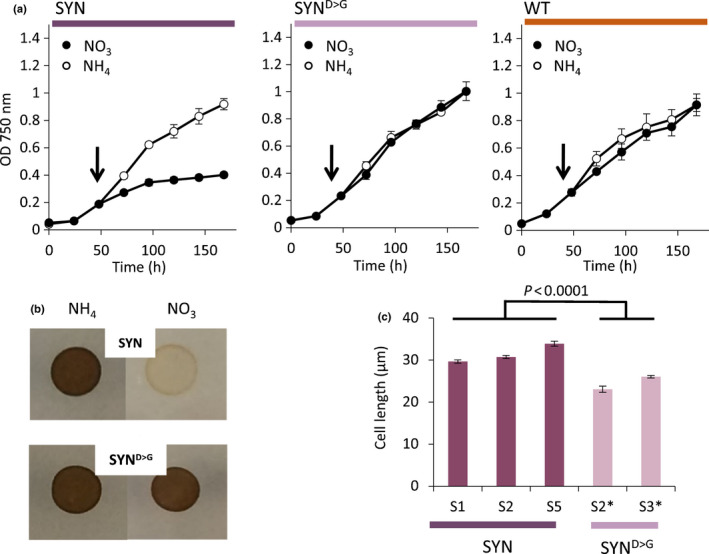
Guanosine penta‐ and tetraphosphate (ppGpp) accumulation affects proliferation and cell elongation. (a) Growth curves of synthetase (SYN), inactive synthetase (SYN^D>G^) and wild‐type (WT) *Phaeodactylum tricornutum* lines. Cells grown inf/2‐NH_4_ were transferred (arrow) to either f/2‐NO_3_ (NO_3_) for induction or f/2‐NH_4_ (NH_4_) as controls. Data are means ± SE of three biological replicates. (b) Cells (SYN, SYN^D>G^) initially grown in liquid f/2‐NH_4_ were grown as cell colonies on agar plates in the presence of either NO_3_ or NH_4_ as controls. (c) Cell length for independent SYN and SYN^D>G^ lines at 5 d post‐induction (±SE, *n* between 60 and 194 cells). No difference in cell width was observed (*P* = 0.059). Statistical significance was calculated using ANOVA with *post hoc* Dunnett test.

We first examined the effects of ppGpp accumulation on growth and proliferation. Five independent SYN lines showed a severe reduction in proliferation upon induction in liquid culture (Figs 2a, S5). By contrast, induction did not affect the proliferation of control SYN^D>G^ lines or the WT. The reduced proliferation of SYN lines was also clearly visible on agar plates following induction (Fig. 2b). Observation by light microscopy showed that proliferation continued slowly up to at least 9 d after induction, with no cell death or damage visible by microscopic observation (Table S3). We also found that SYN cells were significantly longer than those of the controls while maintaining the same width (Fig. 2c). The elongated phenotype of SYN cells may be linked to the reduction in division rate (Fig. 2c).

**Fig. 3:**
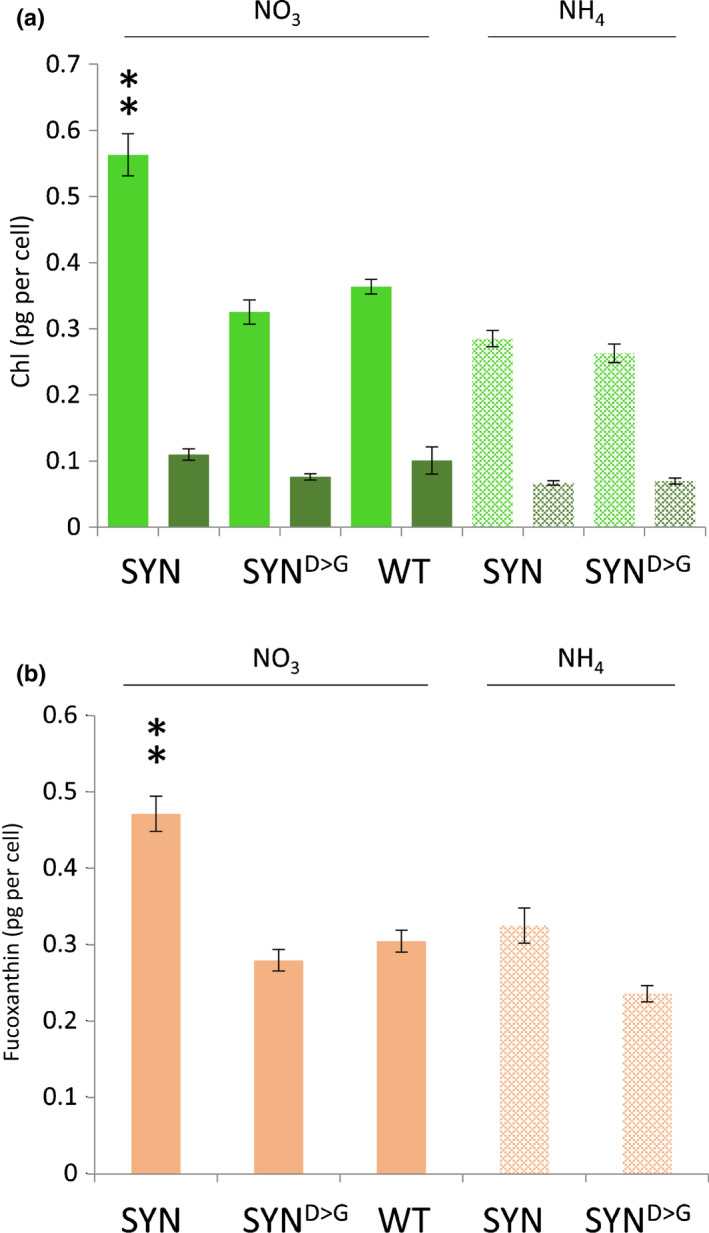
Pigment concentrations are maintained in induced synthetase (SYN) lines. Different *Phaeodactylum tricornutum* lines grown in f/2‐NH_4_ were transferred to inducingf/2‐NO_3_ media (NO_3_) or noninducing f/2‐NH4 media (NH_4_) . Pigments were extracted at 5 d post‐induction, corresponding to the stationary phase of the growth curve, and the concentrations of Chl*a* (light green) and Chl*c* (dark green) (a) and fucoxanthin (b) were determined. Data are means ± SE of five biological replicates. Statistical significance was calculated using ANOVA with *post hoc* Dunnett tests vs wild‐type (WT) control: **, *P* < 0.01.

We then analysed pigment content at different times after induction (Figs 3, S6). In the WT, the concentrations of Chl*a* and fucoxanthin decrease as the cells enter the stationary phase, at 5 d post‐induction. To our surprise, we found that this drop in pigment content does not occur in induced SYN lines: at 5 d after induction, concentrations of Chl*a* and fucoxanthin are significantly higher in SYN than in SYN^D>G^ or the WT (Fig. 3). By contrast, the drop in Chl*c* concentrations was similar in all lines, leading to a higher Chl*a *: Chl*c* ratio in the SYN lines (Fig. 3). These results suggest that ppGpp accumulation inhibits cell division while stabilizing Chl*a* and fucoxanthin concentrations in the chloroplast.

**Fig. 4:**
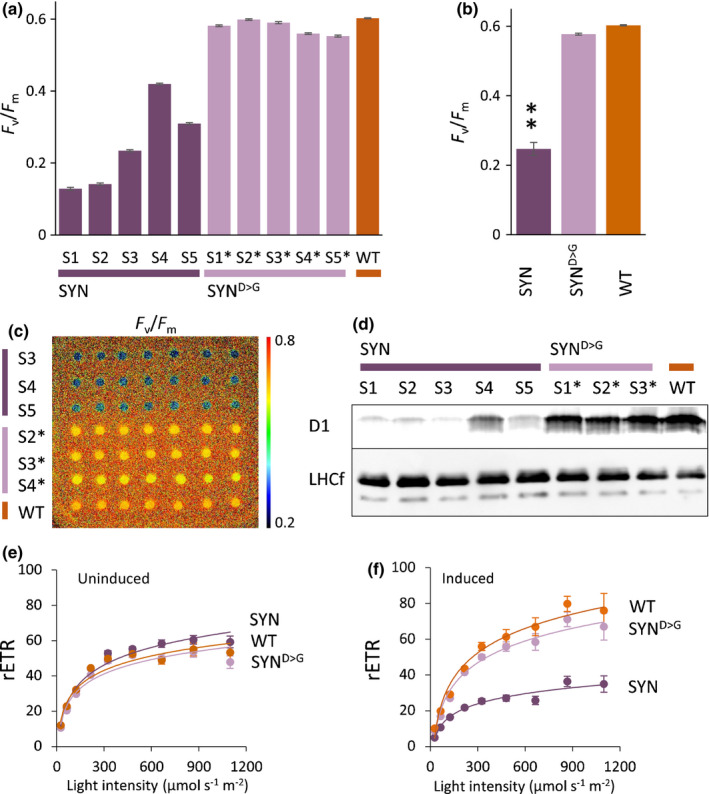
Guanosine penta‐ and tetraphosphate (ppGpp) accumulation downregulates photosynthetic activity in *Phaeodactylum tricornutum*. (a) The maximal efficiency of photosystem II (*F*_v_/*F*_m_) was measured on dark‐adapted cells of independent synthetase (SYN), inactive synthetase (SYN^D>G^) and wild‐type (WT) *P. tricornutum* lines at 2 d post‐induction. Data are means ± SE; *n* = 7 colony spots per line. (b) Average *F*_v_/*F*_m_ of grouped lines for SYN, SYN^D>G^ and WT shown in (a). Statistical significance was calculated using ANOVA with *post hoc* Dunnett tests vs WT control: **, *P* < 0.01. (c) *F*_v_/*F*_m_ false colour image of cell colonies of SYN, SYN^D>G^ and WT at 1 d post‐induction. (d) Immunoblots of protein extracts from equal numbers of cells (7 × 10^4^) at 2 d post‐induction using primary antibodies against D1 and LHCf1‐11. (e, f) Relative electron transfer rate (rETR) at different light intensities in uninduced (e) and induced (f) SYN, SYN^D>G^ and WT *P. tricornutum* lines at 2 d post‐induction. Data are means ± SE for data from grouped lines (five SYN lines, four SYN^D>G^ lines).

In Arabidopsis, ppGpp accumulation has major effects on photosynthetic activity (Maekawa *et al*., 2015; Sugliani *et al*., 2016). We therefore examined the maximal efficiency of PSII (*F*_v_/*F*_m_) (Fig. 4a–c). *F*_v_/*F*_m_ decreased rapidly in induced SYN transformants following induction, reaching a minimum (±SE) of 0.12 ± 0.004 at 2 d post‐induction (Figs 4a,b, S7). The decrease in *F*_v_/*F*_m_ was a result of an increase in basal fluorescence (Fo). By contrast, the *F*_v_/*F*_m_ of the control SYN^D>G^ lines was 0.6 at the same time point after induction. We performed immunoblots to determine whether the decrease in *F*_v_/*F*_m_ in induced SYN lines was a result of changes in PSII architecture. Indeed, we found that the amounts of the chloroplast‐encoded protein D1, a subunit of the PSII reaction centre, decreased in SYN lines (Fig. 4d). By contrast, the total abundances of the light‐harvesting proteins LHCf1–11, which form a major part of the FCP antenna, remained relatively constant (Fig. 4d). These results indicate that the composition of PSII undergoes major changes in response to ppGpp accumulation. In line with the reduced PSII efficiency, the rETR of the entire photosynthetic chain was also lower in SYN lines than in the WT or SYN^D>G^ controls (Fig. 4e,f).

### ppGpp affects the accumulation and distribution of lipids and other reserve compounds

**Fig. 5:**
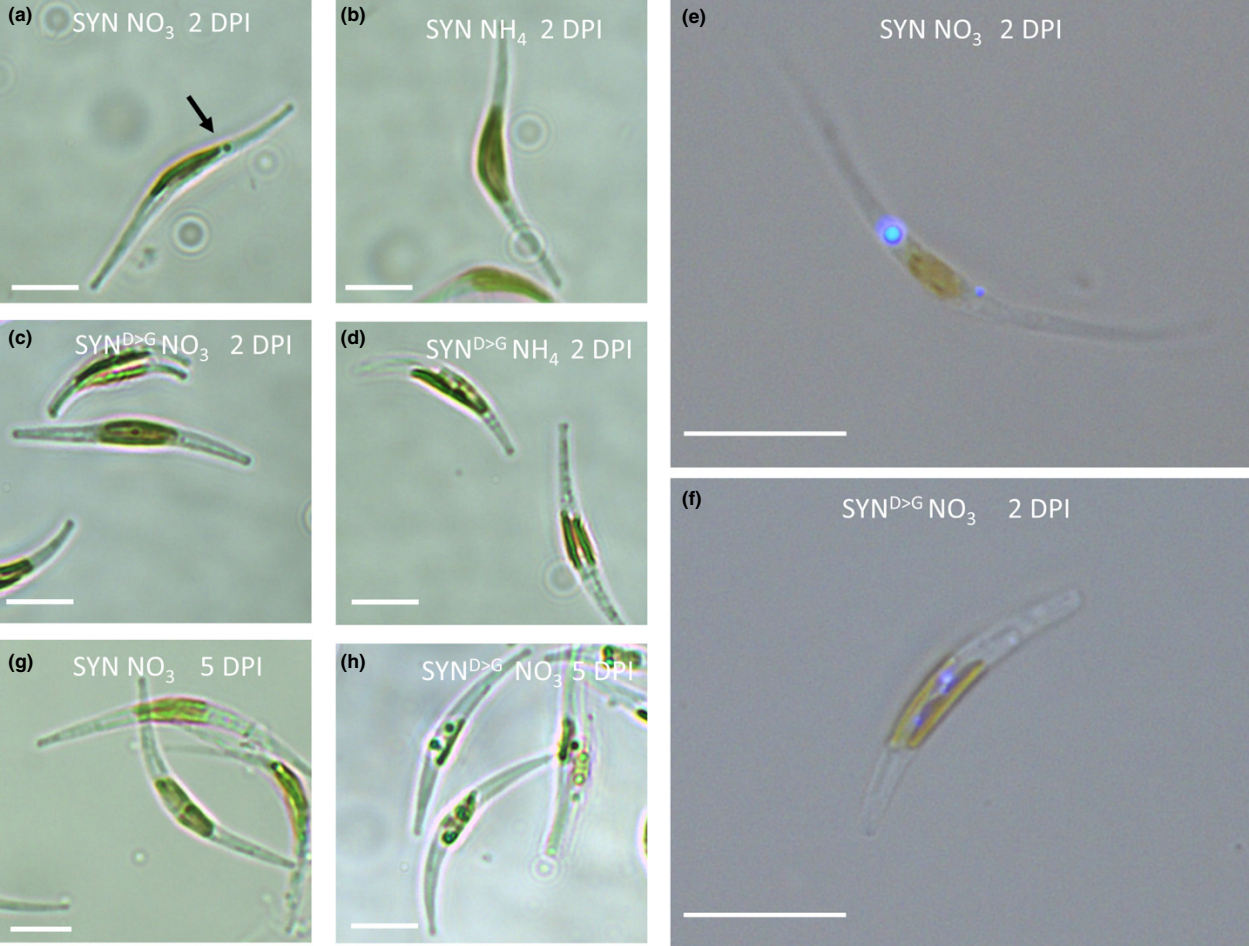
Guanosine penta‐ and tetraphosphate (ppGpp) accumulation affects lipid droplet formation in *Phaeodactylum tricornutum*. (a, b) Induced (a) and noninduced (b) synthetase (SYN) cells after at 2 d post‐induction (2 DPI). (c, d) Induced (c) and noninduced (d) SYN^D>G^ (an inactive mutant form of SYN) cells at 2 d post‐induction. (e, f) Fluorescence microscopy images of SYN (e) and SYN^D>G^ (f) cells at 2 d post‐induction (2 DPI) labelled with the neutral lipid‐specific fluorophore AC202. (g, h) SYN (g) and SYN^D>G^ cells (h) at 5 d after induction (5 DPI). Images are representative of multiple images and at least two independent experimental replicates. Bars, 10 µm.

Using light microscopy, we observed a prominent spot near, but clearly separated from, the chloroplast in all SYN lines at 2 d post‐induction (Fig. 5a). This spot was absent in noninduced SYN (Fig. 5b) and was not detected in SYN^D>G^ lines regardless of the condition (Fig. 5c,d). The prominent spot that was clearly visible under light microscopy was strongly stained by the neutral lipid‐specific fluorophore AC202, indicating that it is a lipid droplet (LD) (Fig. 5e). Observation of the LD by electron microscopy indicated a probable localization within the periplastidial compartment (Fig. S8). AC202 staining revealed the presence of small chloroplast‐associated LDs in the control SYN^D>G^ line (Fig. 5f) and the WT. These droplets differ in size and location from the prominent cytoplasmic LD observed in induced SYN lines and are not visible by light microscopy. The prominent LD in SYN cells disappeared at 5 d after induction (Fig. 5g). This corresponds to the stationary phase of the growth curve, when numerous LDs appear in SYN^D>G^ and the WT (Figs 5h, S9). LDs are indeed well known to accumulate in diatoms with the ageing of the culture (Hu *et al*., 2008).

**Fig. 6:**
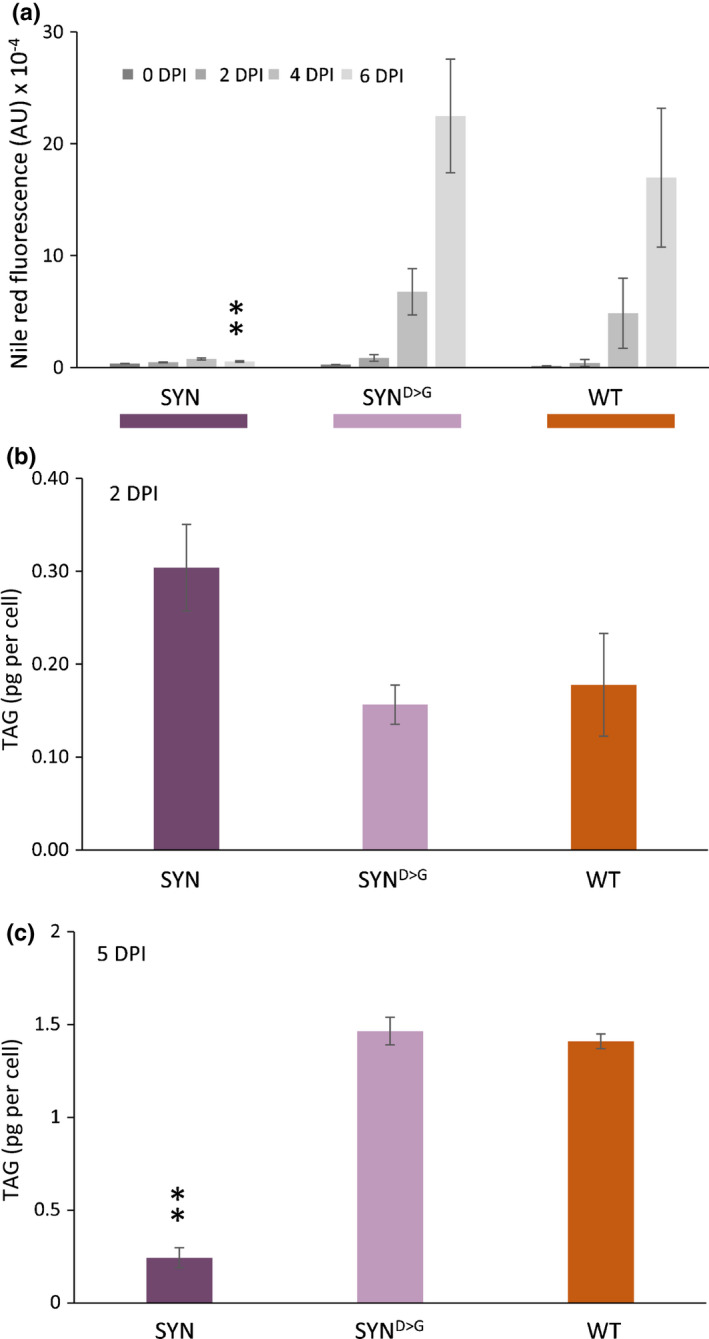
Guanosine penta‐ and tetraphosphate (ppGpp) accumulation inhibits neutral lipid accumulation in the stationary phase. (a) Neutral lipid estimation by Nile red fluorescence in synthetase (SYN), inactive synthetase (SYN^D>G^) and wild‐type (WT) *Phaeodactylum tricornutum* lines at 0, 2, 4 and 6 d post‐induction (DPI). Data are means ± SE of three biological replicates. (b, c) Triacylglycerol (TAG) determination by thin layer chromatography (TLC) at 2 d post‐induction (2 DPI) (b) and 5 DPI (c). Data are means ± SE of five biological replicates. Statistical significance was calculated using ANOVA with *post hoc* Dunnett tests vs WT control: **, *P* < 0.01.

To further characterize the prominent LD in SYN cells, we analysed neutral lipid content at different stages of the growth using flow cytometry and Nile red staining and then confirmed by quantification using TLC (Fig. 6a). A strong correlation between Nile red fluorescence and TAG content is established in diatoms (Greenspan *et al*., 1985; Prioretti *et al*., 2017). Nile red fluorescence, determined using flow cytometry, increased with the age of the culture in WT and SYN^D>G^ cells, but not in SYN cells, in agreement with our microscopic observations (Fig. 5g,h). However, flow cytometry was not sensitive enough to detect differences in lipid content at 2 d post‐induction. Therefore, we directly quantified TAG by extraction of total lipids followed by TLC quantification (Fig. 6b,c). At 2 d post‐induction, the amount of TAG per cell was almost two‐fold higher in the SYN lines than in the controls (*P* = 0.213) (Fig. 6b). Although not significant, this finding tends to support our microscopic observation of higher neutral lipid concentrations in SYN lines at 2 d after induction (Fig. 5a). However, at 5 d post‐induction, SYN lines contained much lower (six‐fold) concentrations of neutral lipids than the control lines, in agreement with the flow cytometry results (Fig. 6c) and microscopy images (Fig. 5g,h). Chrysolaminarin (β‐1,3‐glucan), the main carbohydrate storage compound in diatoms, which is located in the vacuole (Suzuki & Suzuki, 2013), was also less abundant in induced SYN lines than in the controls at 5 d post‐induction (Fig. S10).

**Fig. 7:**
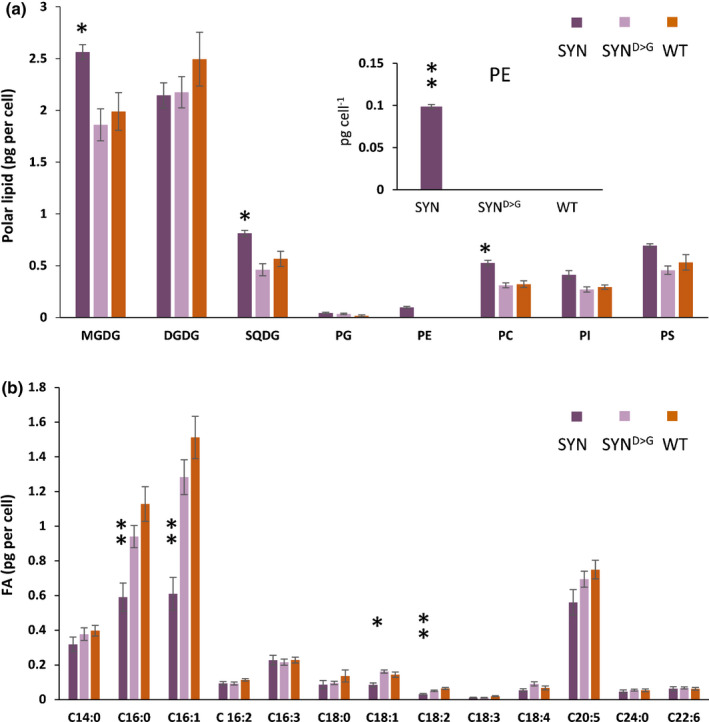
Effects of guanosine penta‐ and tetraphosphate (ppGpp) on polar lipid and fatty acid composition. (a, b) Polar lipid (a) and fatty acid content (b) were determined in synthetase (SYN), inactive synthetase (SYN^D>G^) and wild‐type (WT) *Phaeodactylum tricornutum* lines at 5 d post‐induction. MGDG, monogalactosyldiacylglycerol; DGDG, digalactosyldiacylglycerol; SQDG, sulfoquinovosyldiacylglycerol; PG, phosphatidylglycerol; PE, phosphatidylethanolamine; PC, phosphatidylcholine; PI, phosphatidylinositol; PS, phosphatidylserine. Data are means ± SE of five biological replicates. Statistical significance was calculated using ANOVA with *post hoc* Dunnett tests vs WT control: *, *P* < 0.05; **, *P* < 0.01.

To investigate the impact of altered ppGpp concentrations on membrane lipids, we also analysed polar lipid content. In the membranes of the diatom chloroplast, as in the chloroplasts of other photosynthetic organisms, polar lipids are essentially composed of monogalactosyldiacylglycerol (MGDG), digalactosyldiacylglycerol (DGDG), sulfoquinovosyldiacylglycerol (SQDG) and phosphatidylglycerol (PG) (Abida *et al*. 2015). Polar lipids in SYN cells at 2 d after induction did not significantly differ from those of WT or SYN^D>G^ controls (Fig. S11). However, at 5 d after induction, with the exception of DGDG and PG, the concentration of all the other polar lipids (MGDG, SQDG, phosphatidylcholine (PC), phosphatidylethanolamine (PE), phosphatidylserine (PS)) remained higher in SYN cells than in the SYN^D>G^ and WT controls (Fig. 7a). Furthermore, FA content and composition in SYN cells were different from the controls at 5 d after induction, with substantially lower concentrations of the most abundant FAs (16 : 0 and 16 : 1), and decreases in 18 : 1 and 18 : 2. These changes are consistent with the lower TAG concentrations in SYN lines, because 16 : 0, 16 : 1, 18 : 1 and 18 : 2 are the main FA species found in TAG under nutrient‐limiting conditions in *P. tricornutum* (Abida *et al*., 2015).

### Proteome analysis reveals that ppGpp accumulation leads to the coordinated upregulation of the protein protection response

The analysis of the protein profiles of induced SYN, SYN^D>G^ and WT by SDS‐PAGE revealed major changes in total proteins in SYN lines, including the appearance of additional protein bands (Fig. S2). In order to understand the extent of these changes, we performed an untargeted proteomic analysis to compare protein accumulation between induced SYN lines and induced SYN^D>G^ lines at 2 d after induction. Of the 1046 proteins that were identified, 145 proteins showed a significant difference in accumulation in SYN compared with the control lines (Table S2). The expression of known housekeeping proteins (Siaut *et al*., 2007) was not affected by ppGpp accumulation, with the exception of actin 12 (Table S2). The major groups of proteins showing differential accumulation in SYN are listed in Table 1. Unexpectedly, many chaperones and co‐chaperones showed higher levels of expression in SYN, and the chaperone heat shock protein 20 (HSP20; accession number B7G195) showed the greatest increase. The differentially expressed chaperones in SYN are encoded by nuclear genes and have different predicted cellular localizations. Four chaperones, including HSP40 and one isoform of HSP90, contain the ASAFAP hexapeptide motif that predicts likely chloroplast localization (Gruber *et al*., 2015). Other chaperones have signal peptides for the secretory pathway without an ASAFAP motif, suggesting localization in the endoplasmic reticulum. Some chaperones, such as the Lon protease, which functions as both a chaperone and a protease, are predicted to be mitochondrial.

We found that many transporters and enzymes involved in amino acid metabolism were among those proteins showing the greatest differential accumulation in the SYN lines (Table 1). Notably, the synthesis of glutamate appeared to be upregulated as a result of greater accumulation of the chloroplastic glutamate synthase and glutamate dehydrogenase, and lower concentrations of glutamine synthase. Glutamate is involved in nitrogen assimilation and is also a precursor for Chl biosynthesis. Interestingly, the chloroplast protoporphyrin IX magnesium chelatase, a key enzyme of the Chl biosynthetic pathway, was also more abundant. Proteins of lipid metabolism were also affected. The concentrations of two enzymes involved in FA degradation increased: the mitochondrial acyl‐CoA dehydrogenase (Jallet *et al*., 2020) and a homologue of enoyl‐CoA hydratase, a key enzyme of FA beta‐oxidation. Phage shock protein A, a protein that is involved in managing extra‐cytoplasmic stress responses in bacteria (Joly *et al*., 2010), and glutathione S‐transferase, an enzyme important in the redox homeostasis of the cell, were also more abundant.

**Table 1:** Major groups of proteins showing differential accumulation in response to guanosine penta‐ and tetraphosphate (ppGpp)accumulation in *Phaeodactylum tricornutum*.

Accession number	Description	Log_2_(fold‐change)	Cellular localization
Protein protection, protein quality control and chaperones			
B7G195	HSP20‐like chaperone	8.83	
B7G704	Chaperon ClpB (HSP100)	7.71	
B5Y3Y4	HSP20‐like chaperone	7.15	SP
B7FXQ8	HSP20‐like chaperone	5.98	
B7FUC8	Chaperon ClpB (HSP100)	5.89	
B7G6X2	Von Willebrand factor	5.86	
B7GAC9	Peptidyl‐prolyl cis‐trans isomerase	5.45	
B5Y4C1	HSP20‐like chaperone	5.24	
B7G3Y2	HSP70	5.20	SP
B5Y472	HSP20‐like chaperone	4.96	
B7FSL4	Lon protease	4.71	M
B7G9V1	Von Willebrand factor	4.69	
B7GCE9	HSP70	4.21	SP
B7S4A8	GrpE, HSP90 cofactor	2.83	C
B7FQL4	Armet protein	2.69	
B7FWW7	Heat shock chaperonin‐binding STI1	2.48	C
B7FRV0	Chaperone protein DnaJ (HSP40)	2.39	
B7GEF7	HSP90	2.25	C
B5Y3P1	Chaperone protein DnaJ (HSP40)	2.16	C
Transport			
B7G9Z6	Pyrophosphate‐energized proton pump	4.84	
B7FSN9	Anion‐transporting ATPase	3.90	C
B7FTV6	Nitrate transmembrane transporter	3.85	
B7G5H6	Mitochondrial substrate/solute carrier	3.76	SP
B5Y4J0	ABC transporter	3.18	
B7FQH7	Helical backbone metal receptor (TroA‐like domain)	2.83	C
B7FP05	Xanthine/uracil permease	2.48	
B7GAX8	Nucleotide transporter 1 NTT1	2.13	C
Amino acid metabolism			
B7FPU3	Glutamate synthase	3.71	C
B7G3X3	Glutamate dehydrogenase	3.27	
B7G2T9	Thiamine pyrophosphate enzyme	2.97	C
B7FRL0	Anthranilate synthase	2.96	C
B7G5H9	Aspartate kinase	2.63	C
Chlorophyll synthesis			
B5Y3F4	Protoporphyrin IX magnesium chelatase	2.46	
Redox			
B7G3E0	Glutathione S‐transferase	3.36	
Lipid metabolism			
B7FTR6	Acyl‐CoA dehydrogenase	2.23	M
B7FRZ1	Enoyl‐CoA hydratase	2.86	
Others			
B7G2M8	Phage shock protein A PsP	3.45	
B7S452	Deoxyxylulose‐5‐phosphate synthase	2.67	C
Photosynthesis			
Photosystem II			
A0T0A9	PsbH	−4.87	C*
A0T097	PsbD D2	−3.20	C*
A0T0A3	PsbE	−3.07	C*
A0T096	PsbC	−2.65	C*
A0T0G9	PsbA D1	−2.65	C*
A0T0A4	PsbF	−2.31	C*
B7FZ96	Oxygen‐evolving enhancer protein 1 precursor (PsbO)	−2.05	C*
Photosystem I			
B7GCM3	Flavodoxin	−5.28	C
A0T0A2	Photosystem I assembly protein Ycf4	−3.00	C*
A0T0F3	PsaE	−2.73	C*
Calvin cycle			
B7GE67	FbaC5 fructose‐bisphosphate aldolase class I	−6.34	C
Chloroplastic ribosomal proteins			
A0T0I5	Ribosomal protein S3	−3.65	C*
A0T0C2	Ribosomal protein L11	−3.16	C*
A0T0J8	Ribosomal protein L13	−3.15	C*
A0T0H8	Ribosomal protein L3	−2.94	C*
A0T0I1	Ribosomal protein L2	−2.92	C*
A0T0J9	Ribosomal protein S11	−2.92	C*
A0T0I9	Ribosomal protein L14	−2.60	C*
A0T0J3	Ribosomal protein L6	−2.59	C*
A0T0J1	Ribosomal protein L5	−2.55	C*
Q5D704	Ribosomal protein S16	−2.33	C*
A0T0C7	Ribosomal protein L19	−2.24	C*
A0T0E0	Ribosomal protein S2	−2.20	C*
Fucoxanthin Chl*a*/*c*‐binding proteins			
B7G8Q1	Lhcf15	−3.86	C
B7FR60	Lhcx2	−3.43	C
Transport			
B7S437	Bicarbonate transporter	−6.98	
B7G4H1	Sodium‐dependent phosphate transport protein	−5.37	
B7GCD8	Cation‐transporting P‐type ATPase	−4.14	
B7G0Y4	Ammonium transporter	−4.08	
B7G0V7	Bestrophin chloride channel	−2.91	C
Amino acid metabolism			
B7G5A1	Glutamine synthetase	−2.96	C
B7GB64	Alanine glyoxylate aminotransferase	−2.52	
Others			
B7FYL2	Iron starvation‐induced protein (ISIP2A)	−6.98	SP
B7G9B1	Iron starvation‐induced protein (ISIP2B)	−5.27	SP

C, chloroplast; C*, chloroplast‐encoded; HSP, heat shock protein; M, mitochondria; SP, presence of a signal peptide.

Many chloroplast‐encoded proteins with links to photosynthesis and chloroplast translation were less abundant in SYN. Seven proteins from the PSII complex accumulated to lower levels, including the PsbA/D1 protein, in agreement with our immunoblotting experiments (Fig. 4d). Some proteins from PSI and the Calvin cycle were also less abundant, as well as a bicarbonate transporter and two carbonic anhydrase proteins that were just outside the selection cut‐off (Table S1), together indicating that photosynthetic capacity is diminished at several different levels. The photosynthetic machinery requires large amounts of iron, and consistent with the downregulation of photosynthesis we also observed the downregulation of two iron starvation‐induced proteins. Interestingly, two light‐harvesting antenna proteins, Lhcf15 and Lhcx2, were also less abundant. Therefore, although our immunoblotting experiment showed that total abundances of Lhcf1–11 remained unchanged (Fig. 4d), SYN induction also appears to cause changes in the accumulation of specific antenna isoforms. Also striking was the reduced abundances of 12 chloroplast‐encoded ribosomal proteins in SYN cells, suggesting that ppGpp accumulation has a major effect on chloroplast translation capacity. The ribulose‐1,5‐bisphosphate carboxylase/oxygenase (Rubisco) remained unaltered in our proteomic analysis, as was also directly visible on Coomassie stained protein gels (Fig. S2).

## Discussion

**Fig. 8:**
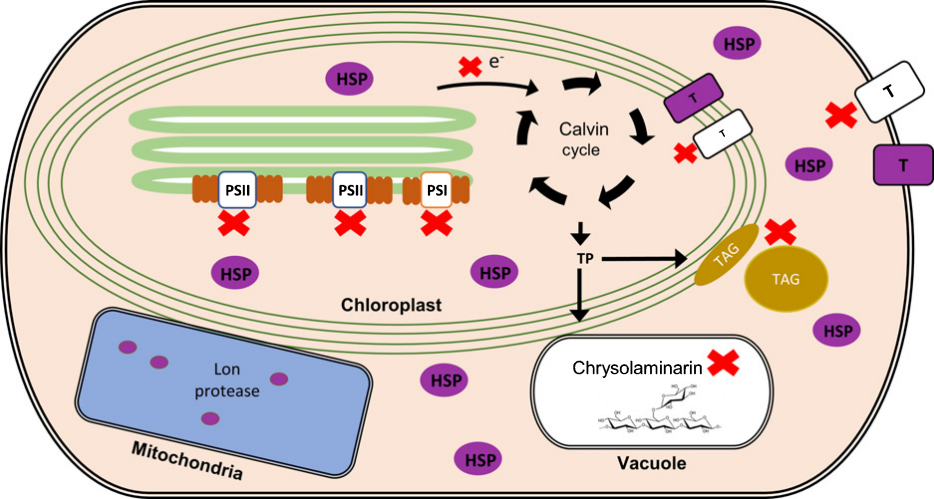
Summary of the effects of guanosine penta‐ and tetraphosphate (ppGpp) accumulation on *Phaeodactylum tricornutum*. ppGpp accumulation causes a decrease in photosynthetic capacity, the chloroplast translation machinery, certain transporters, and the storage molecules triacylglycerol (TAG) and chrysolaminarin (indicated by red ‘X’). At the same time, ppGpp accumulation causes a large increase in the abundance of proteins involved in the protein protection response (shown in purple). TP, triose phosphate; PSII and PSI, photosystems II and I; HSP, heat shock proteins; T, transporters.

In the present work, we studied the function of (p)ppGpp using transgenic *P. tricornutum* SYN lines containing an inducible system for the expression of a bacterial (p)ppGpp synthetase (Fig. 1a). We demonstrate that increased ppGpp concentrations in SYN lines alter the architecture of the photosynthetic machinery and reduce photosynthetic efficiency (Fig. 4). In addition, ppGpp accumulation dramatically decreases the growth rate (Fig. 2), stabilizes the concentration of Chl and fucoxanthin (Fig. 4), and affects accumulation of the reserve molecules TAG and chrysolaminarin (Figs 5, 6, 7, S10, S11). Finally, ppGpp accumulation causes a striking increase in the abundances of chaperones and other stress‐related proteins in multiple cellular compartments (Table 1; Fig. 8).

The independent SYN lines produced different quantities of SYN (Fig. 1). The differences we observed in SYN protein accumulation are probably a result of differences in the copy number or the insertion site(s) of the exogenous DNA in the independent transformants, as previously shown (Falciatore *et al*., 1999). Immunolabelling experiments showed that SYN accumulates preferentially in the chloroplast, further confirming the robust nature of the chloroplast targeting sequence used (Apt *et al*., 2002; Liu *et al*., 2016). Also in support of a principally chloroplastic localization for SYN is the presence of a single band in the immunoblots for SYN, indicating complete processing of the pre‐protein (Fig. 1b), and the strong and specific effects that we observe on chloroplast gene expression that are discussed in more detail later in this section. The average concentrations of ppGpp reached in induced SYN lines (1.3 nmol mg^−1^ dry cell weight) are similar to those reached in *E. coli* (1.2–3 nmol mg^−1^ dry cell weight) during the stringent response (Riesenberg, 1985; Rodionov & Ishiguro, 1995). If we assume that total GTP content is similar between species, then these concentrations of ppGpp (representing 600% of the total GTP) are considerably higher than those observed in Arabidopsis overexpressing a similar (p)ppGpp synthetase (9% of total GTP) (Sugliani *et al*., 2016). This may be a result of differences between unicellular and multicellular organisms, or the capacity of the cell to control ppGpp concentrations. These high ppGpp concentrations did not lead to cell death or complete growth arrest (Fig. 5; Table S3), suggesting that ppGpp does not cause general toxicity. Interestingly, the concentrations of GTP were very similar in all lines, indicating that ppGpp synthesis does not deplete the cellular GTP pool. This result resembles the situation in Arabidopsis ppGpp overaccumulating lines (Bartoli *et al*., 2020) and contrasts with Gram‐positive bacteria, where (p)ppGpp regulates transcription by decreasing the GTP pool in the cell (Krasny & Gourse, 2004).

We also detected ppGpp in WT cells and discovered conditions that cause ppGpp concentrations to increase (Fig. S1), although not to the concentrations measured in SYN lines. Low ppGpp concentrations could be detected in WT cells under normal growth conditions. These ppGpp concentrations correspond to 0.25% of total GTP, which is similar to Arabidopsis where ppGpp accumulates to 0.36% of total GTP under normal growth conditions (Bartoli *et al*., 2020). While nitrogen deprivation did not affect ppGpp concentrations, extended dark treatment caused a four‐fold increase in WT and SYN control lines (Fig. S1). Dark treatment also causes an increase in ppGpp concentrations in Arabidopsis in a Ca^2+^‐dependent RSH (CRSH)‐dependent manner (Ihara *et al*., 2015; Ono *et al*., 2020). However, *P. tricornutum* lacks a direct orthologue of CRSH (Avilan *et al*., 2019), suggesting differences in the underlying mechanism controlling (p)ppGpp synthesis in the dark. The higher concentration of ppGpp observed in SYN lines than in the WT under the conditions tested raises the possibility that concentrations of ppGpp reached in SYN might not occur naturally in diatoms. While the overaccumulation of ppGpp could cause nonphysiological effects, several previous studies have successfully used lines overaccumulating ppGpp to identify targets of ppGpp signalling in plants and bacteria (Schreiber *et al*., 1991; Hesketh *et al*., 2001; Maekawa *et al*., 2015; Sugliani *et al*., 2016). We find that the major effects of ppGpp accumulation in *P. tricornutum* are similar to those in plants (Fig. 4), and that some effects are less severe or absent, such as for Rubisco and Chl loss (Figs 3, 4). Furthermore, while we observe strong effects on growth in *P. tricornutum* (Fig. 2), this does not appear to be attributable to general toxicity, as discussed earlier. Future studies will extend our work by identifying the natural range of ppGpp concentrations, and testing the physiological roles of ppGpp accumulation by the creation of mutants lacking the ability to synthesize ppGpp.

In plants, ppGpp accumulation inhibits photosynthesis, in particular causing a large decrease in the abundance of Rubisco, and in the ratio of PSII reaction centre (RC) to PSII LHC (Maekawa *et al*., 2015; Sugliani *et al*., 2016). Similar results were obtained in this study (Fig. 4). We found that photosynthesis was strongly inhibited in induced SYN lines, and that this was accompanied by a large drop in the PSII RC : FCP antenna ratio and no obvious change in Rubisco concentrations (Fig. 4). Notably, these changes occur despite the significant differences in the structure and organization of the diatom FCP antenna system compared with the equivalent light‐harvesting system in plants (Nagao *et al*., 2019; Pi *et al*., 2019). The drop in PSII RC, and particularly D1, in response to ppGpp accumulation could indicate an increased rate of photoinhibitory D1 destruction. However, the absence of signs of photobleaching or cell death in SYN cells suggests that ppGpp is more likely to act by interfering with D1 synthesis. The overall rate of photosynthesis was also reduced by ppGpp accumulation (Fig. 4). This is consistent with reduced protein abundance across all parts of the photosynthetic machinery, including PSII, PSI, the Calvin cycle and the bicarbonate transport through solute carrier family 4 transporter, SLC4 (Nakajima *et al*., 2013), as well as two carbonic anhydrases involved in the CO_2_‐concentrating mechanism (Tables 1, S1). PSI was less widely affected than PSII, with a particularly strong reduction in concentrations of the PSI acceptor flavodoxin, while in the Calvin cycle there was a large reduction in concentrations of the key Calvin cycle enzyme, fructose bisphosphate aldolase FBAC5 (Gontero *et al*., 2001; Erales *et al*., 2008; Mininno *et al*., 2012). In *P. tricornutum*, FBAC5 is located in the pyrenoid together with two fructose bisphosphate aldolases (Allen *et al*., 2011). Finally, the decreased abundance of a bicarbonate transporter that is likely to supply the Calvin cycle with CO_2_ is reminiscent of the response of cyanobacteria to ppGpp accumulation (Hood *et al*., 2016). Altogether these results indicate that altered PSII architecture and reduced photosynthetic capacity are highly conserved responses to ppGpp accumulation.

In addition to its effects on photosynthesis, one of the most striking consequences of ppGpp accumulation in *P. tricornutum* was the strong induction of a wide range of chaperones and proteases (Table 1). Together these proteins can play an important role in protein protection by preventing protein aggregation and misfolding, and assisting with the refolding or destruction of denatured proteins (Saibil, 2013). This broad upregulation of protein quality control in response to ppGpp accumulation in *P. tricornutum* is highly reminiscent of the chloroplast unfolded protein response (cpUPR) in green algae and plants (Ramundo *et al*., 2014; Ramundo & Rochaix, 2014; Llamas *et al*., 2017; Perlaza *et al*., 2019). The cpUPR can be induced by interference with chloroplast gene expression and leads to the accumulation of small HSPs, chaperones and proteases in the chloroplast and other cellular compartments. Deoxyxylulose‐5‐phosphate synthase (DXS), an enzyme of the chloroplastic isoprenoid pathway, accumulates during the cpUPR (Sauret‐Gueto *et al*., 2006; Llamas *et al*., 2017) and we observe that it also increases in response to ppGpp (Table 1). The induction of the cpUPR along with cpUPR marker proteins strongly suggests that ppGpp inhibits chloroplast gene expression in *P. tricornutum*, as also observed in plants (Maekawa *et al*., 2015; Yamburenko *et al*., 2015; Sugliani *et al*., 2016). The marked reduction in the abundance of chloroplast‐encoded proteins from PSII, PSI, and the translation machinery (Table 1) also supports this, and in the case of the translation machinery is direct evidence of reduced translational capacity.

We also found evidence that ppGpp accumulation may slow cellular ageing in *P. tricornutum* by restricting growth, preventing chloroplast senescence and blocking reserve compound accumulation. ppGpp accumulation caused a striking and sustained inhibition of growth (Fig. 2a) that is considerably stronger than that observed in plants (Maekawa *et al*., 2015; Sugliani *et al*., 2016). It is not currently clear whether the inhibition of growth is a result of the reduction in photosynthetic capacity or potential signalling properties of ppGpp. Shortly after induction of SYN cells, we observed the appearance of a single prominent LD next to the chloroplast, together with a small increase in TAG (Figs 5, 6, S8). The appearance of the prominent LD could be the consequence of a metabolic overshoot following abrupt growth downregulation where, despite the concomitant downregulation of the photosynthetic machinery, the CO_2_ fixation rate still exceeds requirements and the resulting carbon is diverted into the synthesis of reserve compounds. Alternatively, the LD may form as a result of membrane remodelling during photosynthesis downregulation, similar to that previously shown in *P. tricornutum* during nutrient deprivation (Abida *et al*., 2015). In the stationary phase, nutrients become limiting for WT cells and this induces the synthesis of storage compounds and the degradation of photosynthetic pigments. However, we found that ppGpp overaccumulation prevented the degradation of Chl and carotenoids (Fig. 3), and even led to increased concentrations of enzymes for Chl biosynthesis (Table 1). The storage compounds TAG and chrysolaminarin also did not increase in lines overaccumulating ppGpp, and we additionally observed higher concentrations of MGDG and SQDG, consistent with a more stable chloroplast (Figs 5, 6, S10, S11). Together these results suggest that ppGpp overaccumulation prevents cellular ageing by promoting a quiescent‐like state. The anti‐ageing effect of ppGpp in *P. tricornutum* contrasts with the situation in plants where ppGpp accumulation leads to reduced Chl concentrations, reduced chloroplast size and accelerated senescence (Maekawa *et al*., 2015; Sugliani *et al*., 2016). Together these results point to fundamental differences in (p)ppGpp signalling between multicellular and unicellular organisms (i.e. Arabidopsis vs *P. tricornutum*).

The accumulation of ppGpp affects diatom physiology at multiple levels and results in a quiescent‐like state similar to the stringent response in bacteria that helps cells wait out stress conditions (Fig. 8). Reduction of photosynthesis and growth inhibition are common responses to stress in diatoms, suggesting that ppGpp may play an important role in stress acclimation (Litchman *et al*., 2003; Allen *et al*., 2011; Brembu *et al*., 2017). Other cell responses to ppGpp, such as the dramatic increase in HSP20, are also similar to those found in response to dark stress in *P. tricornutum* (Bai *et al*., 2016). However, despite these similarities, the effect of ppGpp accumulation is clearly different from those reported for nutrient stress. For example, under nitrogen starvation, there is a decrease in Chl content and an increase in storage molecules like TAG (Longworth *et al*., 2016). These comparisons suggest that ppGpp signalling may be an important component of a general diatom stress response pathway that acts in concert with other acclimation pathways. Altogether, our findings highlight the importance of ppGpp as a fundamental regulator of chloroplast function across different domains of life, and lead to new questions about the molecular mechanisms and roles of (p)ppGpp signalling in photosynthetic eukaryotes.

## Author contributions

LA, RL, BM, BF and BG conceived and planned the experiments. S Citerne performed the nucleotide quantification; S Cuiné and YL‐B performed and analysed the lipids; LA, CP and BF performed the remaining experiments. LA, BF and BG contributed to the interpretation of the results. LA, BF and BG wrote the manuscript. All authors provided critical feedback and helped to shape the research, analysis and manuscript.

## Supporting information


**Fig. S1** ppGpp concentrations in SYN and wild‐type cells under different conditions.
**Fig. S2** Protein profiles of SYN and controls at 2 d after induction.
**Fig. S3** Volcano plot showing the changes in protein expression at 2 d after induction.
**Fig. S4** SYN is targeted to chloroplasts by the AtpC chloroplast targeting sequence.
**Fig. S5** Growth curves of different SYN and SYN^D>G^ lines.
**Fig. S6** Pigment absorption spectra.
**Fig. S7** Photosynthetic parameters of SYN lines at different time points after induction.
**Fig. S8** Electron micrographs of SYN cells at 2 d after induction.
**Fig. S9** Comparison of lipid droplet phenotypes in SYN^D>G^ and wild‐type cells.
**Fig. S10** Chrysolaminarin concentrations in SYN lines.
**Fig. S11** Effect of ppGpp on polar lipid and fatty acid composition at 2 d post‐induction.
**Methods S1** Expanded methods describing transformation, electron microscopy and proteomics experiments.
**Table S1** List of different primers used in this study.

**Table S2** List of differentially expressed proteins in SYN lines vs controls.
**Table S3** Growth and phenotype of SYN cells during prolonged induction.
Please note: Wiley Blackwell are not responsible for the content or functionality of any Supporting Information supplied by the authors. Any queries (other than missing material) should be directed to the *New Phytologist* Central Office.
