# Supplementary material for: ppGpp influences protein protection, growth and photosynthesis in Phaeodactylum tricornutum
Source: New Phytol. 2021 Mar 19;230(4):1517–32. doi: 10.1111/nph.17286 (PMC8252717; doi:10.1111/nph.17286)
Supplement: Supplementary file 1 — Fig. S1 ppGpp concentrations in SYN and wild‐type cells under different conditions. Fig. S2 Protein profiles of SYN and controls at 2 d after induction. Fig. S3 Volcano plot showing the changes in protein expression at 2 d after induction. Fig. S4 SYN is targeted to chloroplasts by the AtpC chloroplast targeting sequence. Fig. S5 Growth curves of different SYN and SYND>G lines. Fig. S6 Pigment absorption spectra. Fig. S7 Photosynthetic parameters of SYN lines at different time points after induction. Fig. S8 Electron micrographs of SYN cells at 2 d after induction. Fig. S9 Comparison of lipid droplet phenotypes in SYND>G and wild‐type cells. Fig. S10 Chrysolaminarin concentrations in SYN lines. Fig. S11 Effect of ppGpp on polar lipid and fatty acid composition at 2 d post‐induction. Methods S1 Expanded methods describing transformation, electron microscopy and proteomics experiments. Table S1 List of different primers used in this study. [file NPH-230-1517-s002.pdf]

New Phytologist

New Phytologist **Supporting Information**
Article title: **ppGpp influences protein protection, growth and photosynthesis in *Phaeodactylum tricornutum***
Authors: Luisana Avilan, Regine Lebrun, Carine Puppo, Sylvie Citerne, Stephane Cuiné, Yonghua Li-Beisson, Benoit Menand, Ben Field, Brigitte Gontero
Article acceptance date: 08 February 2021

The following Supporting Information is available for this article:

Methods S1 Expanded methods describing transformation, electron microscopy and proteomics experiments.
Fig. S1 ppGpp levels in SYN and wild type cells under different conditions.
Fig. S2 Protein profiles of SYN and controls two days after induction.
Fig. S3 Volcano plot showing the changes in protein expression two days after induction.
Fig. S4 SYN is targeted to chloroplasts by the AtpC chloroplast targeting sequence.
Fig. S5 Growth curves of different SYN and SYN$^{D>G}$ lines
Fig. S6 Pigment absorption spectra.
Fig. S7 Photosynthetic parameters of SYN lines at different time points after induction.
Fig. S8 Electron micrographs of SYN cells two days after induction.
Fig. S9 Comparison of lipid droplet phenotypes in SYN$^{D>G}$ and wild type cells.
Fig. S10 Chrysolaminarin levels in SYN lines.
Fig. S11 Effect of ppGpp on polar lipid and fatty acid composition two days post induction.

Table S1 List of different primers used in this study.
Table S2 List of differentially expressed proteins in SYN lines versus controls (separate file).
Table S3 Growth and phenotype of SYN cells during prolonged induction.

Methods S1 Expanded methods describing transformation, electron microscopy and proteomics experiments.

Transformation of *P. tricornutum*

Transformation of *P. tricornutum* was performed using a Bio-Rad Biolistic PDS-1000/He particle delivery system with rupture disc of 1350 psi as previously described (Falciatore *et al.*, 1999; Kroth, 2007). Prior to the bombardment, 1 x 10^7 cells, in the exponential growth phase, were spread in the center (5 cm diameter) of the f/2-NH4 agar plate without antibiotic, dried under sterile hood and placed under illumination for 24 h. Ten µl of M17 tungsten microcarriers (1.1 µm diameter, Bio-Rad) equivalent to 600 µg particle were coated with 1 µg of the specific plasmid constructs in the presence of $CaCl_2$ and spermidine (Kroth, 2007) and used to bombard the diatom cells. After 24 h illumination cells were resuspended and re-plated onto f/2-NH_4 agar plates containing 80 µg mL^{-1} zeocin (Invitrogen). The plates were maintained under illumination for 3 weeks and the resistant colonies were screened by PCR for the presence of *SYN* genes as previously described (Falciatore *et al.*, 1999) (primers in Table S1). A cell suspension from each colony was re-plated on agar medium and the screening process was repeated. The final colonies were also screened for the presence of the proteins SYN by Western blotting. Interestingly, we obtained fewer SYN lines (n=9) than $SYN^{D>G}$ (n=66), suggesting that the presence of SYN negatively affects the recovery of transgenics. Clones of *SYN* lines were prone to losing their phenotype after several multiplications. The transformants were therefore periodically re-plated from cells stored at 4°C and re-screened based on their growth phenotype on solid agar plates.

Electron microscopy and immunogold labelling

Cells were fixed by directly adding the fixative into the culture medium (final concentration: 0.25% glutaraldehyde, 1% paraformaldehyde). After 1h at the culture temperature, the cells were placed at 4 °C. The following day the cells were washed 3 times in PHEM buffer 1.5x + 9% sucrose (Montanaro *et al.*, 2016) and post-fixed in 1% osmium tetroxide (EMS 19150) for 1 h at 4 °C. Samples were washed again in distilled water and treated with 1% uranyl acetate (EMS 22400) for 1 h at 4 °C in the dark. Subsequently samples were dehydrated in an acetone series and embedded in Epon resin (EMS). Ultrathin sections (60 – 90 nm) were cut, stained with uranyl acetate and lead citrate and analyzed using a Tecnai 200KV electron microscope (FEI). Images were acquired with a digital camera (Eagle, FEI).

For immunolabelling experiments cells were high pressure frozen (Leica EMPACT 2), freeze substituted (Leica AFS 2) and embedded in Epon resin as recently described (Dell'Aquila *et al.*, 2020). Immunolabelling was performed on ultrathin 50 nm sections. Sections were prepared by incubating briefly (1-3 min) in a saturated solution of sodium metaperiodate, rinsing in TBS with 1% Triton X-100 for 5 min, and blocking for 1 h in blocking solution (5% BSA, 0.1% fish skin gelatin). Sections were then incubated overnight with the primary antibody (anti-RelA) at a 1/200 dilution in Tris buffered saline (TBS) for 1 h, rinsed 4 times with TBS for 5 min, and then incubated

for 1 h at 37°C with anti-rabbit antibodies conjugated to 6 nm gold particles (Aurion, GAR-90604/2) at a 1/30 dilution in TBS. Finally, sections were rinsed 4 times with TBS for 5 min, and incubated in 2.5% glutaraldehyde in 0.05 M sodium cacodylate for 10 min. Immunolabelled sections were then stained with lead citrate and observed with a FEI Tecnai G2 electron microscope. Images were acquired with a digital camera (Velata, Olympus).

Mass spectrometry analysis

Peptides were separated on an analytical C18 column by a two step -linear gradient from 4% to 20% of mobile phase B (0.1% (vol/vol) formic acid (FA)/ 80% (vol/vol) acetonitrile) in mobile phase A (0.1% (vol/vol) FA) for 90 minutes, then from 20% to 45% of B in A for 30 minutes. For peptide ionization in the nanosource spray, voltage was set at 1.65 kV and the capillary temperature at 275 °C. Top 10 Data Dependent workflow was used in a 400-1600 m/z range and a dynamic exclusion of 60 s.

Spectral data were processed for protein identification and quantification using the MaxQuant computational proteomics platform (version 1.6.5.0) integrating the search engine Andromeda and the MaxLFQ algorithm (Cox *et al.*, 2014). Spectra were searched against a UniProt *P. tricornutum* database (date 2018.01; 10715 entries) supplemented with a set of 245 frequently observed contaminants. The search parameters were set at: (*i*) trypsin cleavage authorized before proline with two missed cleavages allowed; (*ii*) monoisotopic precursor tolerance of 20 ppm in the first search used for recalibration, followed by 4.5 ppm for the main search and 0.5 Da for fragment ions from MS/MS; (*iii*) cysteine carbamidomethylation (+57.02146) as a fixed modification and methionine oxidation (+15.99491) and N-terminal acetylation (+42.0106) as variable modifications; (*iv*) a maximum of five modifications per peptide allowed and (*v*) minimum peptide length was 7 amino acids and a maximum mass of 4,600 Da. The false discovery rate (FDR) at the peptide and protein levels were set to 1% and determined by searching a reverse database. Statistical analysis was performed with Perseus (version 1.5.6.0) (Tyanova *et al.*, 2016). LFQ normalized intensities were uploaded from the proteinGroups.txt and converted to base 2 logarithms to obtain a normal distribution. Quantifiable proteins were defined as those detected in at least 70% of samples in at least one condition. Missing values were replaced using data imputation by randomly selecting from a normal distribution centred on the lower edge of the intensity values. To determine whether a given detected protein showed differential accumulation, a two-sample t-test was applied using a permutation-based FDR set at a conservative threshold of 0.0001 (250 permutations), and the p value was adjusted using a scaling factor s0 set to 1.

Cox J, Hein MY, Luber CA, Paron I, Nagaraj N, Mann M. 2014. Accurate proteome-wide label-free quantification by delayed normalization and maximal peptide ratio extraction, termed MaxLFQ. *Mol Cell Proteomics* **13**: 2513-2526.

Dell'Aquila G, Zauner S, Heimerl T, Kahnt J, Samel-Gondesen V, Runge S, Hempel F, Maier UG. 2020. Mobilization and cellular distribution of phosphate in the diatom *Phaeodactylum tricornutum*. *Frontiers in Plant Science* **11**: 579.

Falciatore A, Casotti R, Leblanc C, Abrescia C, Bowler C. 1999. Transformation of nonselectable reporter genes in marine diatoms. *Mar Biotechnol (NY)* **1**: 239-251.

Kroth PG 2007. Genetic transformation: a tool to study protein targeting in diatoms. In: Giezen vd ed. *Protein Targeting Protocols. 2 ed. Methods in Molecular Biology*. Totowa, NJ Humana Press Inc., 257-269.

Montanaro J, Gruber D, Leisch N. 2016. Improved ultrastructure of marine invertebrates using non-toxic buffers. *PeerJ* **4**: e1860.

Tyanova S, Temu T, Sinitcyn P, Carlson A, Hein MY, Geiger T, Mann M, Cox J. 2016. The Perseus computational platform for comprehensive analysis of (prote)omics data. *Nature Methods* **13**: 731-740.

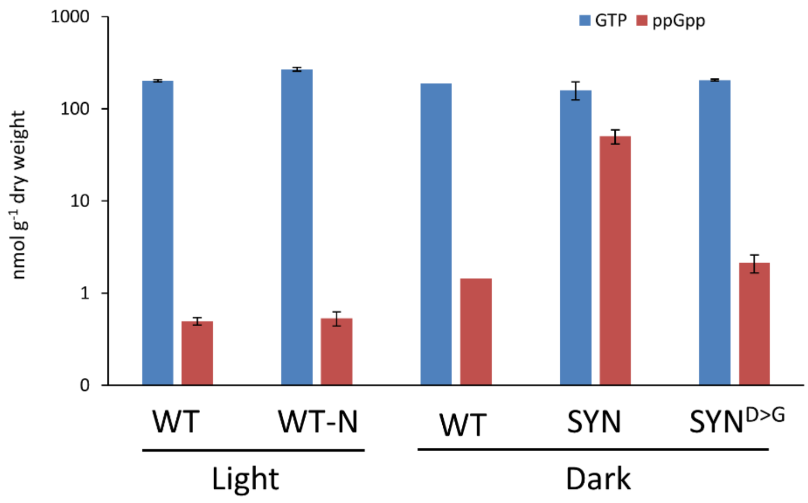

Fig. S1. ppGpp levels in SYN and wild type cells under different conditions. ppGpp and GTP levels in SYN (±SE, n=5 independent lines), SYN$^{D>G}$ (±SE, n =4 independent lines) and WT (1 replicate) 21 days post induction with incubation in the dark, and in WT cultured in the light in standard f/2-NO_3 medium or f/2 medium without nitrogen (-N) (±SE, n= 3 biological replicates).

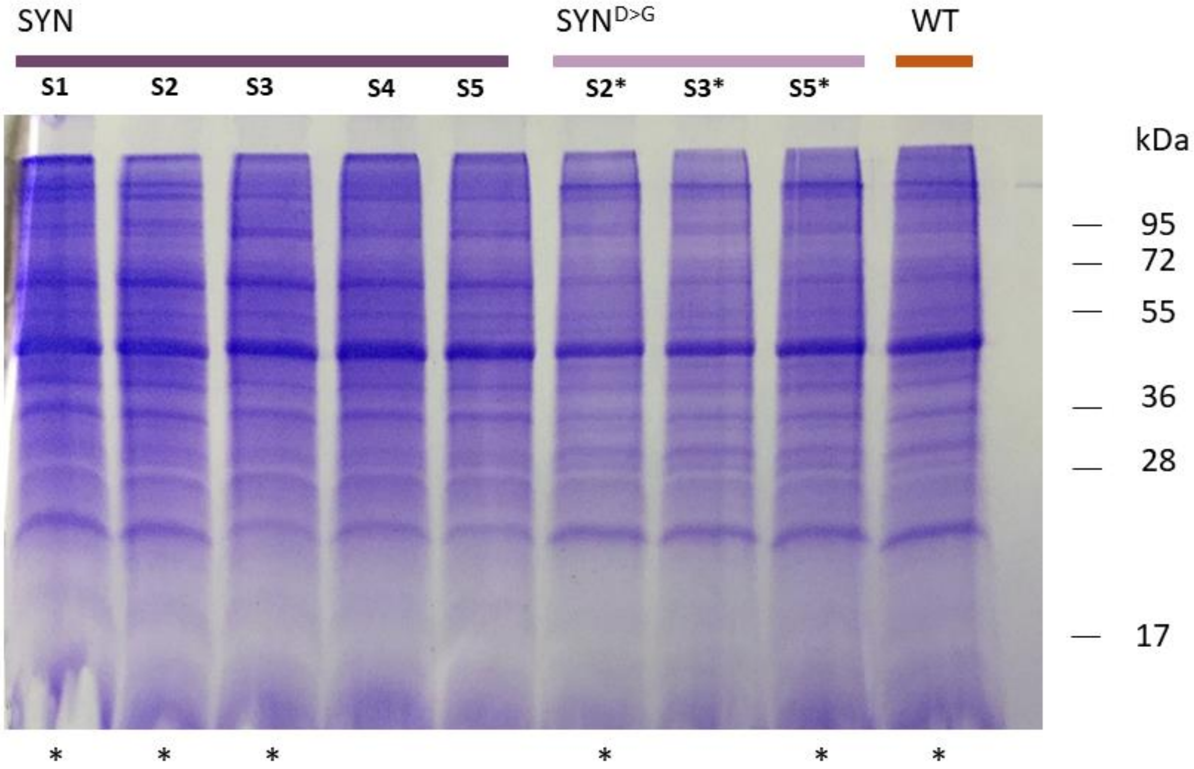

Fig. S2. Protein profiles of SYN and controls two days after induction. Protein extracts (50 µg protein) from several induced SYN and $SYN^{D>G}$ lines and the WT, were separated by SDS-PAGE and stained with Coomassie Brilliant Blue. Stars below the gel indicate the samples used for proteomic analysis.

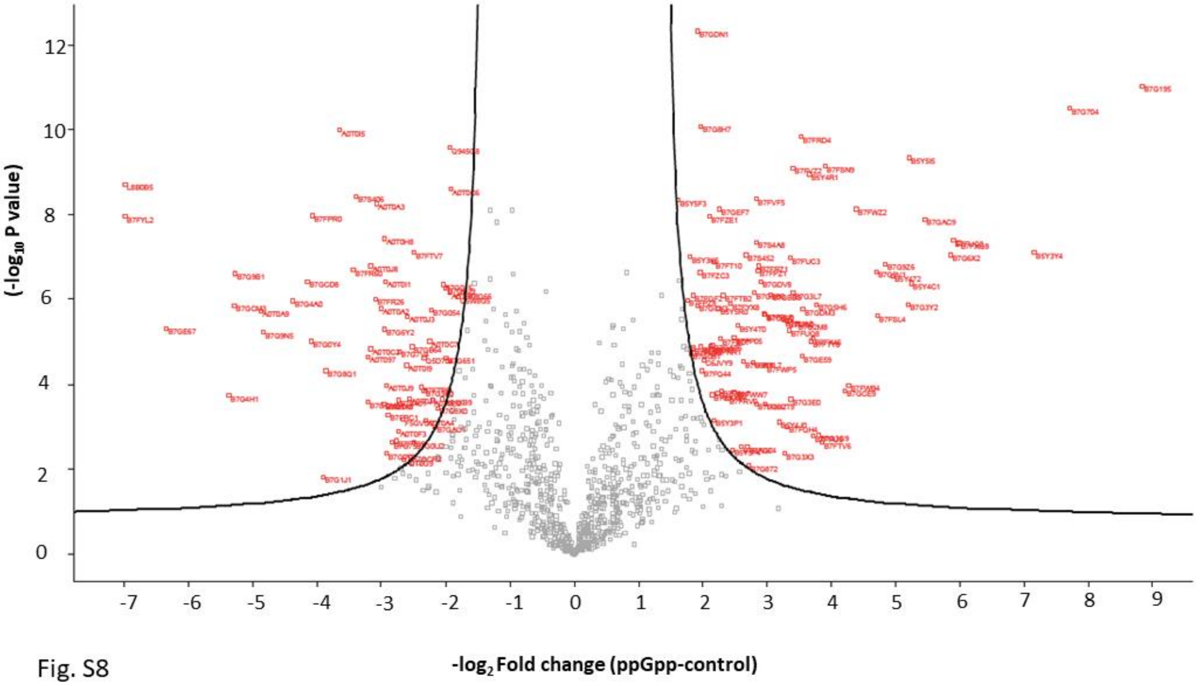

Fig. S8

Fig. S3. Volcano plot showing the changes in protein expression two days after SYN induction. The plot highlights the differentially accumulating proteins in SYN lines. The x-axis shows $\log_2$ fold change of control ($SYN^{D>G}$ and WT) versus SYN. The y-axis shows the log10 p value. Proteins with a p value < 0.1 and a fold change of 4 are highlighted in red.

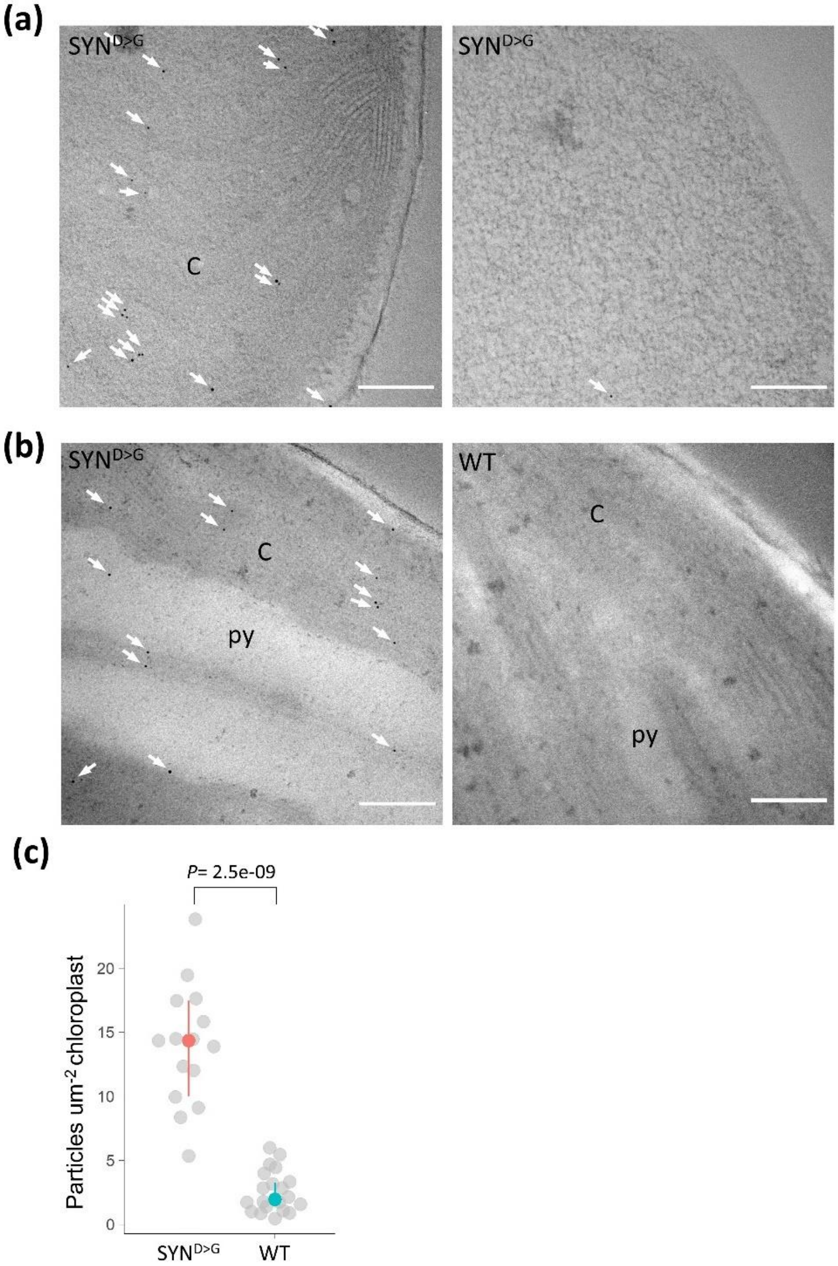

Figure S4. SYN is targeted to chloroplasts by the AtpC chloroplast targeting sequence. (a-c) Immunogold labelling on sections from SYN$^{D>G}$ and WT cells 2 days post induction was used to confirm targeting of SYN to chloroplasts by the AtpC chloroplast targeting sequence. (a) Immunogold particles targeting SYN (indicated by white arrows) accumulated preferentially in the chloroplast (left panel) compared to other parts of the same cell (right panel). (b) Immunogold particles targeting SYN were more abundant in the chloroplasts of SYN expressing cells than WT cells. (c) Immunogold particle density in chloroplasts was significantly higher in SYN$^{D>G}$ than in WT (Wilcoxon test, n=15-20 chloroplasts from independent cells). Mean, 95% confidence intervals and individual data points (grey) shown. Scale bars, 200 nm; C, chloroplast; py, pyrenoid.

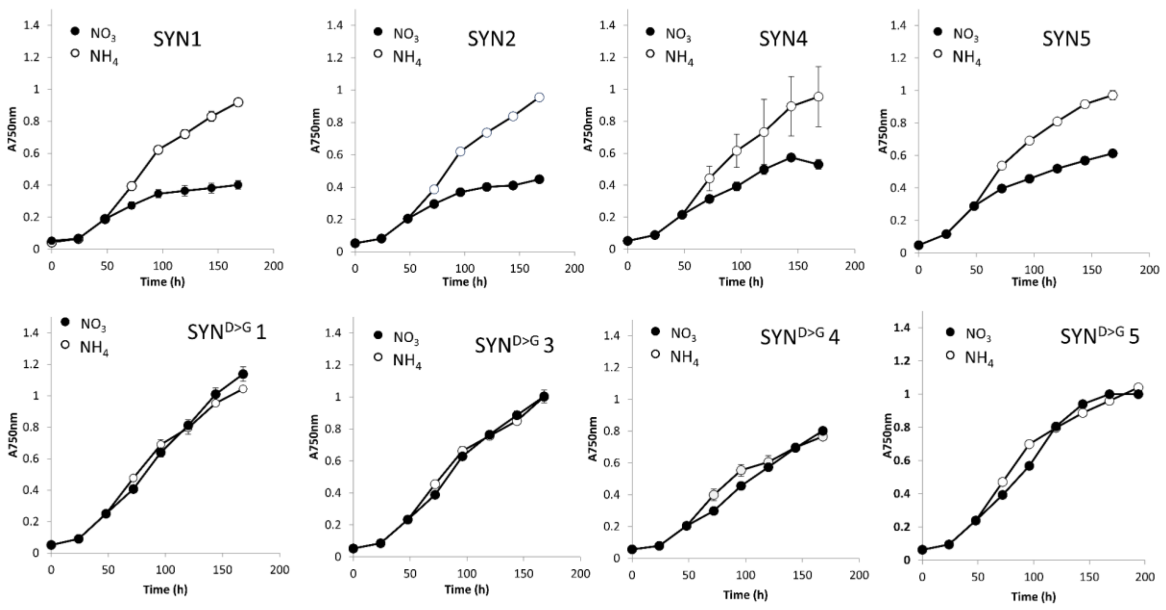

Fig. S5. Growth curves of different SYN and SYN$^{D>G}$ lines. Cells grown in f/2-NH_4 were transferred after two days to either f/2-NO_3 for induction or f/2-NH_4 for the non-induced control. Data are means ± SE of three biological replicates.

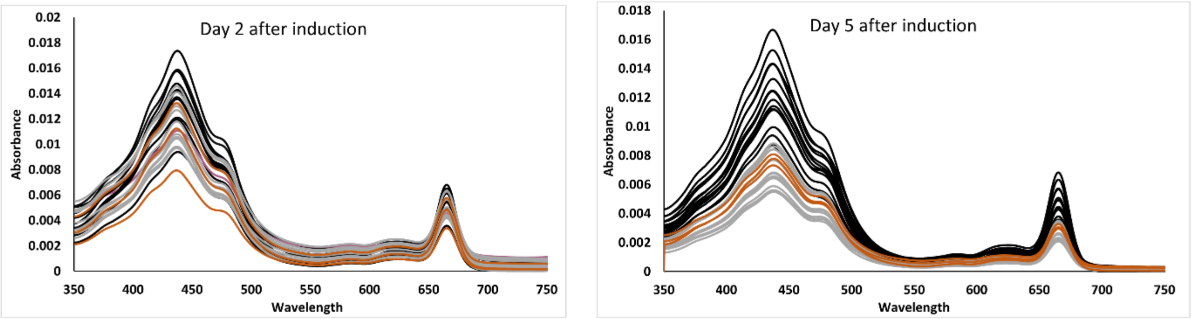

Fig. S6. Pigment absorption spectra. Pigments from SYN (black), $SYN^{D>G}$ (gray) and WT (brown) cells were extracted with ethanol. Cells were grown in f/2-NH_4 and transferred to f/2-NO_3 for induction and harvested two and five days post induction.

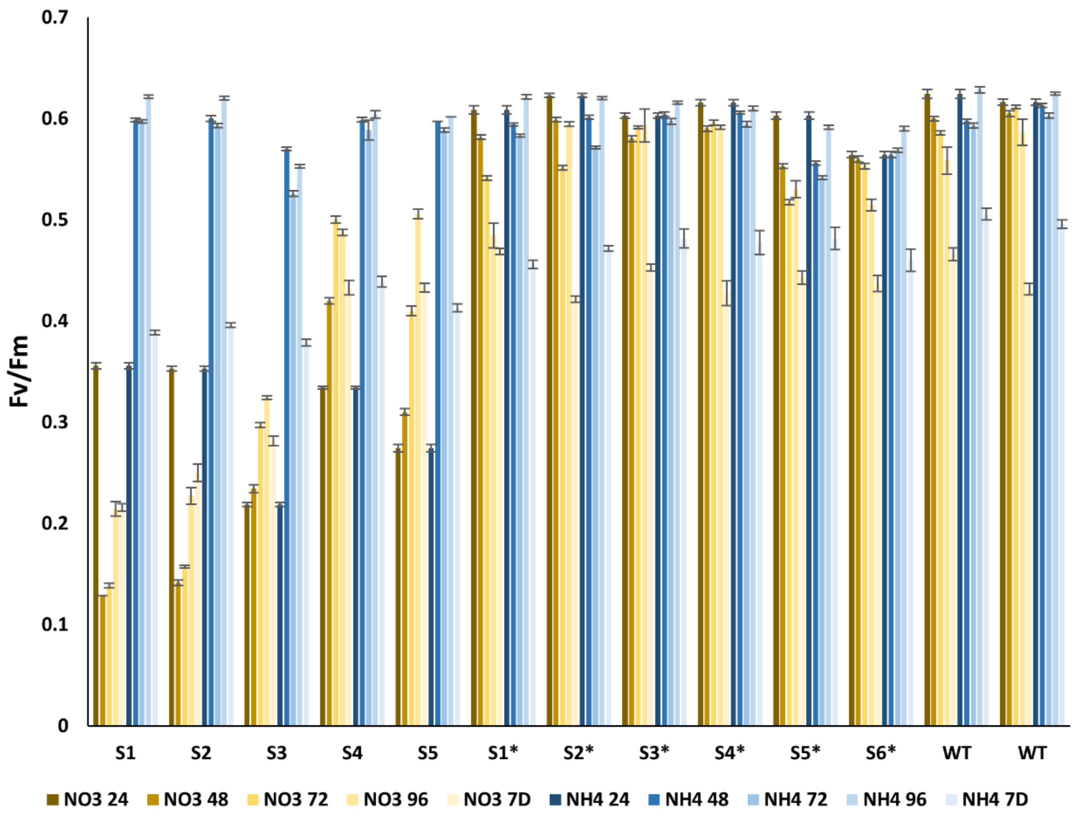

Fig. S7. Photosynthetic parameters of SYN lines at different timepoints after induction. The maximal efficiency of the photosystem II (F_v/F_m) was measured at 24, 48, 72, 96 hours and 7 days post induction with f/2-NO_3 (NO3), in five independent SYN lines (S1-S5), six independent $SYN^{D>G}$ lines (S1*-S6*) and WT. Controls were cultures grown in in f/2-NH_4 (NH4). Averages are shown, ± SE, n=7.

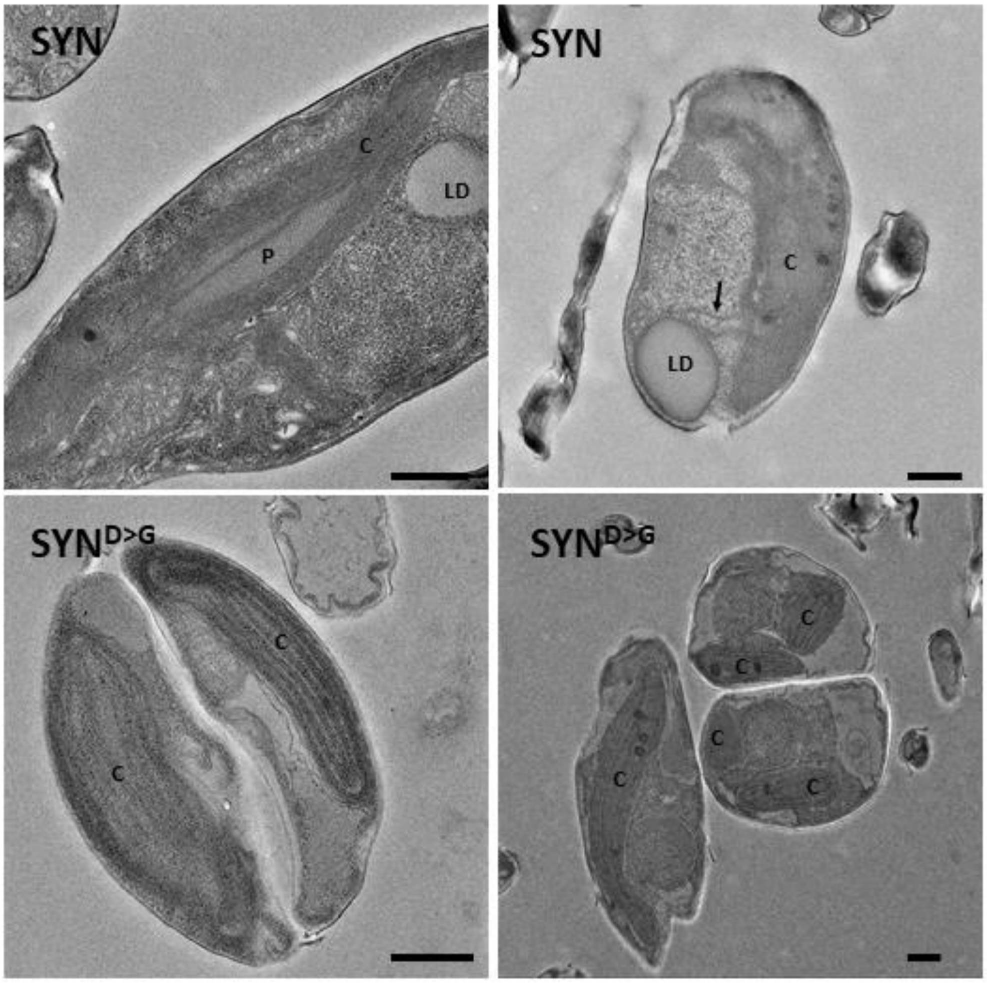

Fig. S8. Electron micrographs of SYN cells two days after induction. SYN and SYN$^{D>G}$ cells were prepared and imaged by transmission electron microscopy two days after induction. Representative images of SYN and SYN$^{D>G}$ cells are shown. Large lipid droplets (LD) were observed only in SYN cells, and often appeared surrounded by a second membrane system linked to the chloroplast (arrow) suggesting localisation within the periplastidial compartment. C, chloroplast. Scale bars, 500 nm.

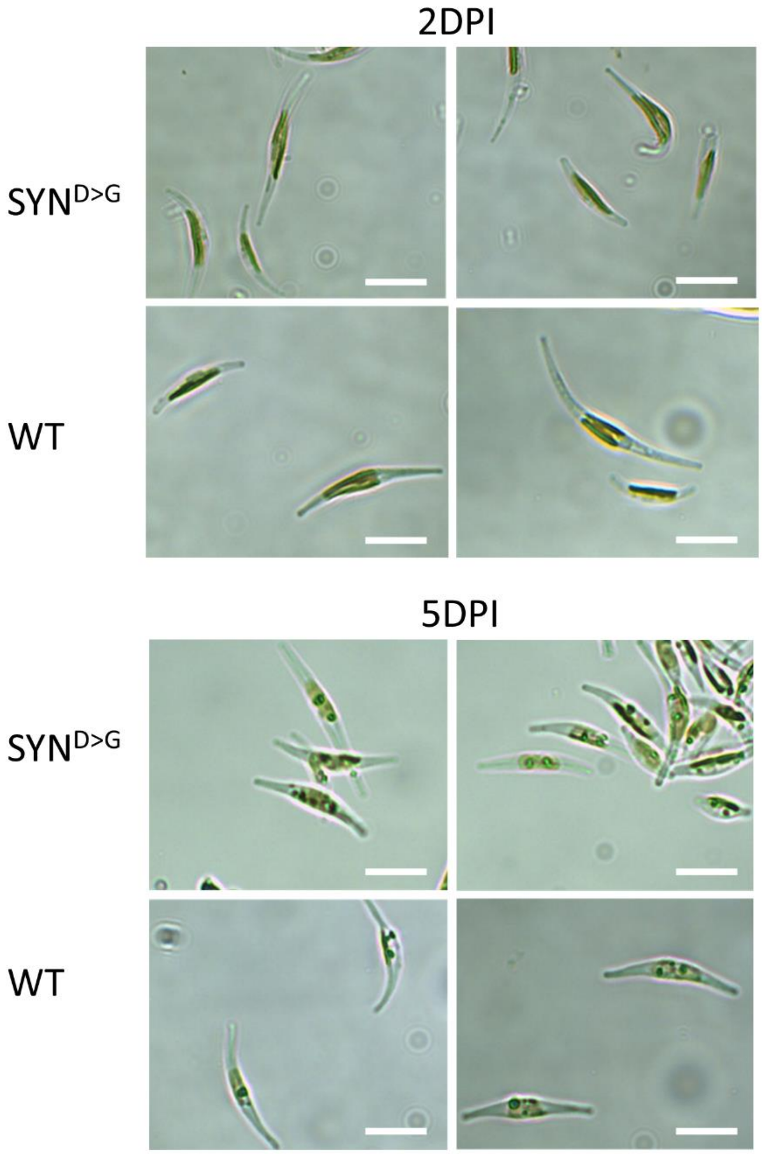

Fig. S9. Comparison of lipid droplet phenotypes in SYN$^{D>G}$ and wild type cells. SYN$^{D>G}$ and wild type (WT) cells two and five days after induction (2 DPI, 5 DPI). The scale bar corresponds to 10 µm.

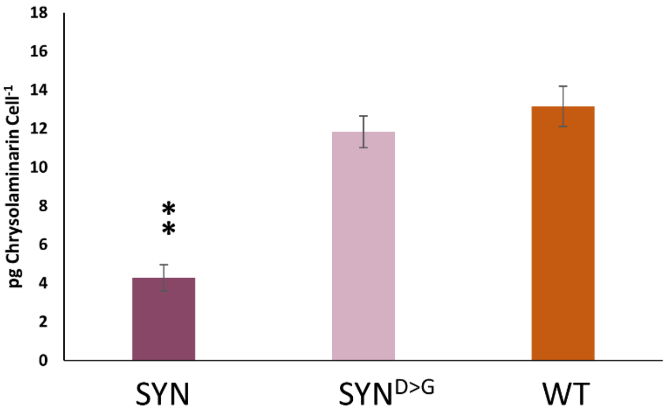

Fig. S10. Chrysolaminarin levels in SYN lines. Chrysolaminarin levels were determined five days post induction in cell pellets containing 2 x 10^7 cells. Data are means ± SE of five biological replicates, analysed by ANOVA using Dunnett post-hoc test versus WT control, **P < 0.01.

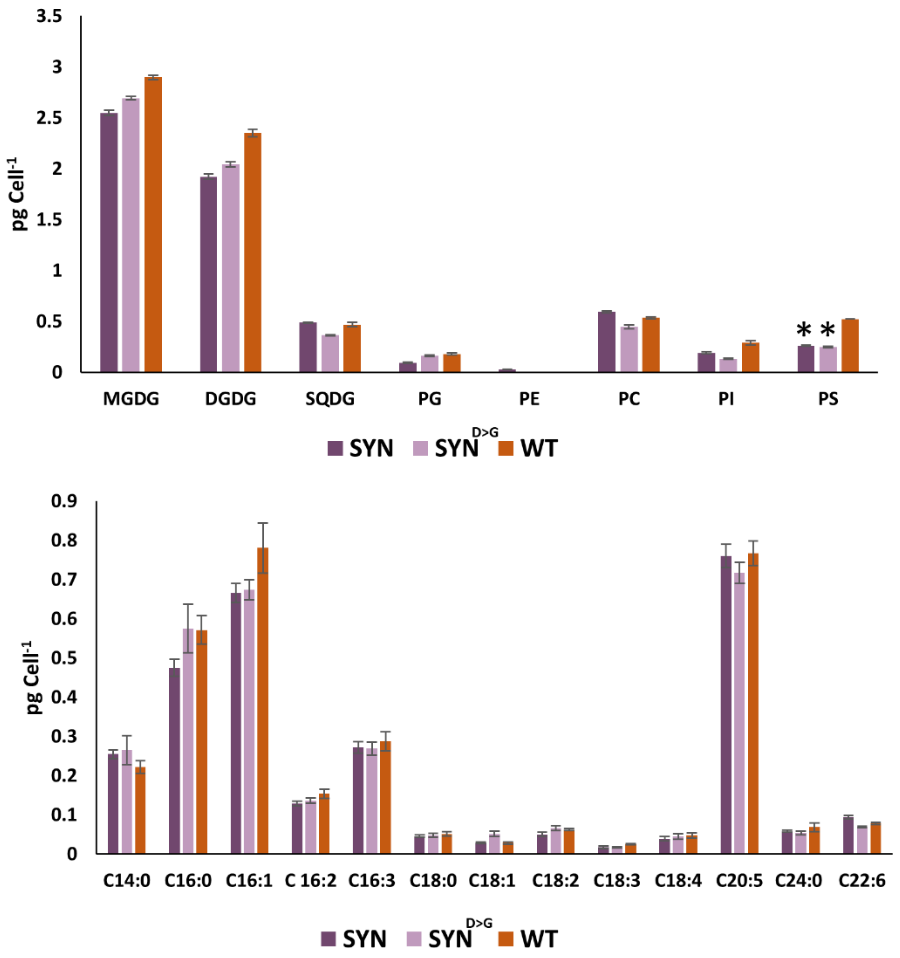

Fig. S11. Effect of ppGpp on polar lipid and fatty acid composition two days post induction. Polar lipids (upper panel) and fatty acid levels (lower panel) were determined two days post induction. MGDG, monogalactosyldiacylglycerol; DGDG, digalactosyldiacylglycerol; SQDG, sulfoquinovosyldiacylglycerol; PG, phosphatidylglycerol; PE, phosphatidylethanolamine; PC, phosphatidylcholine; PI, phosphatidylinositol and PS, phosphatidylserine. Data are means ± SE of five biological replicates, analysed by ANOVA using Dunnett post-hoc test versus WT control, *P<0.05, **P < 0.01.

Table S1. **List of primers used in this study**

Name	Sequence (5’-3’)	Experiment
ATPc-up	CACTTGTGCGAACGGAATTCAAGATGAGAT CCTTTTGCATCGC	Amplification of chloroplastic gamma ATP synthase bipartite targeting peptide and cloning
ATPc-Syn-low	TTACCGCAAC CATGACAATCGTTGCTTTACG	Amplification of chloroplastic gamma ATP synthase bipartite targeting peptide and cloning
Syn-up	GATTGTCATG GTTGCGGTAAGAAGTGCACA	Amplification of SYN and cloning
Syn- low	CTTAAAGTAAATTGAAGCTTTTAATGGTGAT GGTGATGGT	Amplification of SYN and cloning
ATP-Syn-up	ATGAGATCCTTTTGCATCGCAGC	Screen PCR
ATP-Syn-low	AATGGTGATGGTGATGGTGTCCA	Screen PCR
Sh-ble-up	TCGAGTTCTGGACCGACCGGCT	Screen PCR
Sh-ble-low	ACGAAGTGCACGCAGTTGCCGG	Screen PCR

Table S3. **Growth and phenotype of SYN cells during prolonged induction.**

	Cell/ml X 10^5	
**Day 1**		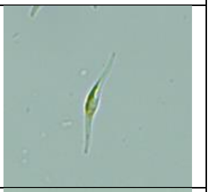
**Day2**	23.58	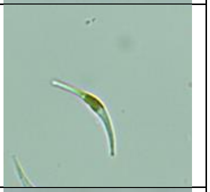
**Day 3**		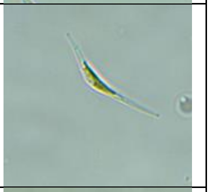
**Day 4**		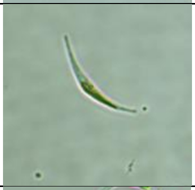
**Day 5**	38	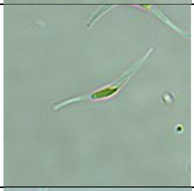
**Day 6**		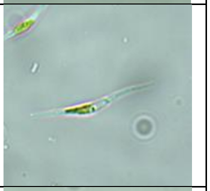
**Day 7**	43.6	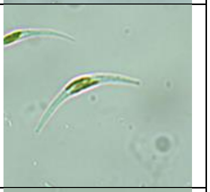
**Day 8**		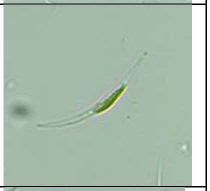
**Day 9**	50	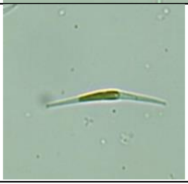
